# Effects of Bioactive Compound Supplementation on Insulin Resistance and Systemic Inflammation in Overweight and Obese Adults: A Systematic Review and Meta-Analysis of Randomized Controlled Trials

**DOI:** 10.3390/nu18213638

**Published:** 2026-10-31

**Authors:** Sarah Nichele, Lucas Moneró Prates, Luca Busetto

**Affiliations:** 1 Department of Land, Environment, Agriculture and Forestry, Università degli Studi di Padova, Viale dell’Università 16, 35020 Legnaro, Italy; 2 Department of Statistics, Università di Bologna, Via Belle Arti, 41, 40126 Bologna, Italy; lucas.moneroprates@studio.unibo.it; 3 Department of Medicine (DIMED), Università degli Studi di Padova, Via Niccolò Giustiniani 2, 35028 Padova, Italy; luca.busetto@unipd.it

**Keywords:** bioactive compounds, supplementation, insulin resistance, inflammation, obesity treatment, systematic review

## Abstract

**Background/objectives**: Obesity is characterized by chronic low-grade inflammation and insulin resistance. This systematic review and meta-analysis evaluated oral supplementation with curcumin, green tea extract, resveratrol and omega-3 fatty acids on homeostatic model assessment of insulin resistance (HOMA-IR) and C-reactive protein (CRP) in adults with overweight or obesity. **Methods**: PubMed and Scopus were searched on 22 July 2026 for randomized controlled trials (RCTs) of isolated oral supplementation versus placebo or control, lasting ≥4 weeks, in adults with BMI ≥25.0 kg/m^2^ without type 2 diabetes. Risk of bias was assessed with RoB 2 and certainty of evidence with GRADE. Random-effects meta-analyses were performed separately for each compound and biomarker, using mean difference (MD). **Results**: Fifty-nine RCTs (3263 participants) were included (omega-3: 23; curcumin: 13; green tea: 13; resveratrol: 10). Most trials had some concerns or high risk of bias. Only curcumin significantly reduced HOMA-IR (MD = −0.30 [−0.53, −0.07], *p* = 0.019; moderate certainty), with low heterogeneity. Green tea extract yielded a non-significant CRP reduction (MD = −0.43 mg/L [−0.87, 0.02], *p* = 0.057; low certainty) with substantial heterogeneity. Resveratrol and omega-3 showed no significant effect on either outcome (low certainty). Significant heterogeneity was observed in five of eight analyses. **Conclusions**: Curcumin may modestly improve insulin resistance in this population, potentially as a complementary strategy in obesity management. Green tea’s anti-inflammatory effect requires confirmation. Heterogeneous formulations and doses, small samples and risk of bias limit interpretation; future RCTs should use standardized formulations with documented bioavailability.

## 1. Introduction

Obesity is one of the most prevalent chronic diseases worldwide, affecting more than 1 billion individuals. Projections indicate that, by 2050, more than 3.8 billion people will be living with overweight or obesity, making it one of the most significant global public health challenges [1]. Beyond being a major risk factor for the development of conditions such as type 2 diabetes, cardiovascular diseases, fatty liver disease, certain types of cancer, and dementia [2], obesity is currently recognized as a chronic, relapsing, and progressive disease characterized by dysfunctional adipose tissue expansion and systemic metabolic impairment [3].

The excessive expansion of adipose tissue does not represent a passive energy storage phenomenon, but rather a biologically active process that profoundly reconfigures the secretory profile of the tissue [4]. Under normal weight conditions, adipocytes perform essential endocrine functions, secreting anti-inflammatory adipokines such as adiponectin, which promote insulin sensitivity and exert cardioprotective effects [5]. With dysfunctional adipose tissue expansion; however, this profile is substantially altered: adiponectin production is suppressed, while the release of pro-inflammatory cytokines such as tumor necrosis factor-alpha (TNF-α), interleukin-6 (IL-6), and interleukin-1 beta (IL-1β) is increased [6]. These cytokines, once released into systemic circulation, reach the liver and stimulate hepatocytes to produce acute-phase proteins, most notably C-reactive protein (CRP) [7]. The persistent elevation of CRP, even at modest levels characteristic of low-grade inflammation, reflects the continuous activation of the innate immune response that accompanies obesity, and serves as a measurable indicator of the metabolic dysfunction established, underpinning obesity’s classification as a metabolic-inflammatory disease [3]. This inflammatory environment, combined with increased free fatty acid release from lipolysis and oxidative stress [8], activates stress-sensitive intracellular pathways (such as the c-Jun N-terminal kinase (JNK) and nuclear factor kappa B (NF-κB) pathways) that interfere with insulin signaling and lead toward a progressive impairment of tissue capacity to respond adequately to insulin. Insulin resistance does not represent merely an intermediate marker of metabolic risk, but a condition with broad clinical and functional implications. Its persistence is associated with the development of type 2 diabetes mellitus, metabolic syndrome, cardiovascular diseases, and non-alcoholic fatty liver disease [2,3]. Together, these phenomena sustain a cycle of metabolic worsening in which chronic inflammation and insulin resistance reinforce one another, making the disruption of this cycle a central therapeutic objective in obesity management [9,10].

In this context, weight loss remains the primary strategy for reversing the metabolic dysfunctions associated with obesity. Reduction in adipocyte volume promotes normalization of the adipose tissue secretory profile, with restoration of adiponectin levels, reduction in pro-inflammatory cytokines, and consequent improvement of insulin sensitivity [11]. Current clinical guidelines recommend lifestyle interventions (dietary modification, physical activity and psychological support) as the pillars of treatment, with pharmacological or surgical options reserved for cases of greater clinical complexity [11]. However, the effectiveness of weight loss as an isolated strategy faces significant biological and behavioral limitations. It is estimated that only a minority of individuals with obesity are able to maintain long-term weight loss without continuous support [12]. This scenario highlights that weight loss, while desirable and clinically relevant, cannot be the sole therapeutic horizon. Therefore, identifying complementary strategies capable of attenuating metabolic dysfunction, with or in the absence of weight reduction, represents a significant clinical advance for the large proportion of the population that lives with obesity throughout their lives.

In this regard, a question of growing relevance emerges in the literature: could naturally occurring food-derived bioactive compounds such as polyphenols, flavonoids, and essential fatty acids be capable of mitigating the effects of chronic low-grade inflammation and improving insulin sensitivity, independently of significant weight loss? The hypothesis that bioactive compounds could help restore partially or completely tissue function in the low-grade chronic inflammation state of obesity finds initial support in a robust body of evidence from pre-clinical studies. In animal models, particularly rodents with diet-induced obesity, specific bioactive compounds have demonstrated the capacity to reduce inflammatory mediators by modulating metabolic signaling pathways or interest [13].

Resveratrol, for example, has demonstrated the ability to activate sirtuin 1 (SIRT1) and AMP-activated protein kinase (AMPK) pathways in adipose and skeletal muscle tissue, promoting reduced expression of pro-inflammatory cytokines, decreased NF-κB activation, and improved insulin sensitivity in diet-induced obesity models [14,15,16]. Curcumin, in turn, suppresses NF-κB activation in adipose tissue, reducing local TNF-α and IL-6 production, attenuating pro-inflammatory macrophage recruitment, and improving the glycemic profile in murine models of obesity and diabesity [17]. Green tea extracts, a category that encompasses several preparations of Camellia sinensis leaves rich in catechins such as epigallocatechin gallate (EGCG), have demonstrated in experimental models the capacity to reduce intracellular lipid accumulation, attenuate systemic inflammatory markers, and decrease fasting serum glucose and insulin resistance in rodents fed a high-fat diet [18,19]. Omega-3 fatty acids, although a class of bioactive lipids rather than a single compound, through the production of resolvins and protectins, can actively promote the resolution of inflammation in adipose tissue, restore key mediators involved in insulin signaling (such as insulin receptor substrate 1 and 2 (IRS-1/IRS-2) and glucose transporter type 4 (GLUT-4)) and improve insulin tolerance in animal models of obesity [20,21].

This body of evidence not only demonstrates the existence of biologically plausible effects but establishes sufficiently mechanistic pathways to justify the translation of these hypotheses into clinical trials. On this basis, bioactive compounds have been investigated as intervention strategies for improving glycemic control and reducing systemic inflammation in individuals with overweight and obesity [8,13,16,22,23]. When translated into clinical trials in humans, some studies suggest promising effects; however, results are heterogeneous and at times contradictory [24,25]. Previous meta-analyses have examined these compounds individually on inflammatory and glycemic markers but have done so predominantly in populations defined by conditions such as metabolic syndrome, polycystic ovary syndrome, non-alcoholic fatty liver disease or cardiometabolic disease [26,27,28,29,30,31,32,33,34,35]. No quantitative synthesis has yet estimated and compared the effects of these four compounds on the same outcomes, within a population defined primarily by excess adiposity and under a single analytical framework. Given all the statements presented before, the present systematic review and meta-analysis of randomized controlled trials (RCTs) aims to fill this gap, answering the following research question: does supplementation with bioactive compounds (here included resveratrol, curcumin, green tea extracts, and omega-3 fatty acids) reduce systemic inflammation and improve insulin resistance in adults with overweight or obesity, in the presence or absence of associated inflammatory metabolic conditions?

## 2. Materials and Methods

This systematic review was conducted and reported in accordance with the Preferred Reporting Items for Systematic Reviews and Meta-Analyses (PRISMA) 2020 statement [36]. The protocol was registered in PROSPERO (registration number CRD420261453236). The selected bioactive compounds were chosen based on their established relevance in suppressing inflammatory responses in pre-clinical studies and their potential role in improving insulin signaling, as identified through a preliminary review of the existing literature.

The population was restricted to adults with overweight or obesity (body mass index, BMI ≥ 25.0 kg/m^2^). Participants with diagnosis of diabetes were excluded to minimize the interference of a chronic underlying disease and related pharmacologic management on the outcomes. Pregnant women, oncological patients, and athletes were also excluded, as these conditions involve metabolic alterations that deviate from the expected basal metabolism of the target population. Interventions were limited to compound supplementation to control for at least 4 weeks. Consequently, studies involving interventions with dietary patterns, mixed compounds (such as formulations with Curcumin + Resveratrol or Resveratrol + vitamins), or whole food matrices (e.g., Mediterranean diet or food-source interventions) were excluded. We use “bioactive compound” as an umbrella term for these four interventions, even though omega-3 fatty acids are, strictly speaking, a group of bioactive lipids rather than a single compound. The green tea category included both green tea extracts and the administration of isolated catechins or epigallocatechin gallate (EGCG); the specific formulations are detailed in Supplementary Files S2 and S3. Studies without proper control or placebo group were also ineligible. We included RCTs with either parallel or crossover designs.

As primary outcomes, the HOMA-IR was selected as the glycemic marker due to its wide clinical validation as a reliable surrogate for insulin sensitivity in epidemiological and clinical settings. CRP was chosen as the primary inflammatory marker due to its highly sensitive systemic indicator of low-grade chronic inflammation associated with adipose tissue expansion. Secondary outcomes included fasting glucose, fasting insulin, glycated hemoglobin (HbA1c), TNF-α, adiponectin, and IL-6, which, although included in the search, are not addressed in the present work. Studies were ineligible where neither primary outcome was measured, or where an outcome was measured but the results were not reported in a form permitting the calculation of an effect size. No restriction was applied to the year of publication. PICOS framework is presented in Table 1.

The systematic literature search was performed on 22 July 2026, across PubMed and Scopus databases, via the NCBI interface and the Elsevier platform, respectively. The search strategy was constructed using a combination of Medical Subject Headings (MeSH) terms and free-text keywords (title and abstract) for specific databases. Boolean operators (AND, OR, NOT) were used to cross-reference terms related to the population (obesity/overweight), interventions (bioactive compounds), and outcomes (insulin resistance and inflammatory markers). The detailed search strings for each database are provided in Table 2.

The selection process was conducted in consecutive stages, with screening, full-text assessment and data extraction of the included studies conducted by a single reviewer (S.N.). The extracted data were checked against the original articles by L.M.P., and decisions on protocol-level exceptions to the eligibility criteria and on the clinical plausibility of reported values were taken by the second reviewer (L.B.).

Reasons for exclusion during the full-text phase included an ineligible population, lack of a proper control group, insufficient statistical data (i.e., missed or unclear information, absence of the primary outcomes), use of whole food matrices, use of combined interventions (e.g., resveratrol + green tea) and unavailability of the full text. Data from the included studies were extracted into a standardized spreadsheet and are presented in Appendix A (Appendix A.1, Appendix A.2, Appendix A.3 and Appendix A.4). The complete dataset entered the meta-analyses, including the conversion procedures applied, is provided in Supplementary File S1. Detailed eligibility criteria are presented in the Supplementary Materials (Table S1).

### 2.1. Risk of Bias

Risk of bias was assessed for each outcome of each trial using RoB 2 [37] for the effect of assignment to intervention, with its crossover version for crossover trials [38], based on the published article and, when available, the trial protocol or registry record. Trials were classified as low risk, some concerns or high risk. Domain 5 was rated as low risk when no pre-specified plan was accessible but there was no sign of selective reporting. The certainty of the evidence for each outcome was assessed using the GRADE approach [39] and rated as high, moderate, low or very low. Both assessments were performed by two researchers (S.N. and L.M.P.), and disagreements were resolved by discussion. Risk of bias was rated by the share of the pooled weight from trials at each RoB 2 level (not serious if ≥50% low risk; very serious if ≥50% high risk; otherwise, serious), and borderline cases were stated explicitly. The assessment is available in Supplementary Files S2 and S3. Inconsistency was judged from *I*^2^, confidence-interval overlap and the prediction interval. Imprecision was judged using the confidence-interval approach [40], relating the 95% CI to a threshold of 0.2 SD of the control group, taken as a small effect; the optimal information size (800 participants for this effect) was considered only when the CI did not cross the threshold and the effect was large. Publication bias was assessed visually on funnel plots and did not lead to downgrading; no outcome was upgraded. These rules were defined by the authors post hoc. The summary of findings is presented in Section 3 (Results), and the full evidence profiles are provided in the Supplementary File S4.

### 2.2. Quantitative Analysis

The meta-analyses were conducted using JASP (Version 0.99.0) with the Classical Meta-Analysis module. For each bioactive compound (curcumin, green tea extract, resveratrol, and omega-3 fatty acids), separate meta-analyses were performed for each primary outcome (HOMA-IR and CRP), yielding eight independent pooled analyses. Effect sizes were computed using the independent groups design with quantitative measurement. The mean difference (MD) was chosen as the effect size measure, as it preserves the clinical interpretability of the results, although it requires a common unit of measurement across studies. All transformations were applied consistently across trials. The values entered each model, together with the conversion method applied in each case, are reported on a study-by-study basis in Supplementary File S1, organized by compound and outcome.

A random-effects model was fitted using restricted maximum likelihood (REML) estimation of the between-study variance (τ^2^), given the methodological heterogeneity across studies, including differences in population characteristics, intervention dose and duration, and outcome measurement protocols. Statistical significance of the pooled effect was evaluated using the Knapp–Hartung adjustment, given the small number of included studies. Between-study heterogeneity was quantified using the Q statistic, I^2^ and τ, and 95% prediction intervals were reported alongside confidence intervals (CI).

Two categories of exclusion were applied. First, trials reporting clinically implausible values were discarded prior to model fitting, as determined by L.B.; this was the case for Campbell et al. (2019) [41], whose fasting insulin values yielded a derived standard deviation exceeding the group mean, suggestive of extreme outliers or a reporting error. Second, following the initial model fit in JASP, asymmetry in the residual funnel plot was inspected visually to assess the potential for small-study effects, and entries identified as outliers were removed for the performance of a sensitivity analysis. An entry was pre-established as an outlier if it fell entirely outside the 95% pseudo-confidence region of the residual funnel plot; borderline entries lying at the immediate boundary of the region were retained. Funnel plot inspection was conducted jointly by two reviewers (S.N. and L.B.); no formal statistical test of funnel plot asymmetry was performed.

Trials with more than one active arm compared with a single placebo group [42,43,44]. contributed one comparison per active arm, and the shared placebo group was divided equally among them. For crossover trials, the standard error of the mean difference was multiplied by √(1 − r) to approximate a paired analysis, assuming r = 0.5 [45,46].

## 3. Results

The initial database search identified 1496 records (PubMed: *n* = 452; Scopus: *n* = 1044). After applying automated filters, 774 records remained. Following the removal of 140 duplicates, 634 unique articles were screened, and 507 were excluded based on title and abstract.

Of the 127 reports sought for retrieval, 11 could not be obtained (full text unavailable). The remaining 116 reports were assessed for eligibility, of which 57 were excluded: ineligible population (*n* = 3); lack of an appropriate control or placebo group (*n* = 5); insufficient numerical data or failure to report the pre-specified primary outcomes (*n* = 25); use of food matrices (*n* = 12); combined interventions (*n* = 10); and overlapping reports (*n* = 2). A total of 59 studies were included. A flow diagram of study identification, screening, and inclusion is reported in Figure 1.

A total of 59 reports, representing 59 unique RCTs, were included in this meta-analysis. The trials were categorized by bioactive compounds: Omega-3 (*n* = 23 studies), Curcumin (*n* = 13), Green Tea (*n* = 13), and Resveratrol *(n* = 10). The Summary of Included Studies is presented in Appendix Table A1, Table A2, Table A3 and Table A4, and a more detailed version is available in the Supplementary File S5. Age and BMI are reported as in the original values and units. Metabolic status was also recorded, as the included populations extended beyond overweight or obesity, the primary inclusion criteria, to encompass individuals with varied inflammatory and metabolic conditions, such as polycystic ovary syndrome (PCOS), metabolic syndrome, and non-alcoholic fatty liver disease (NAFLD). Given that such conditions may differentially affect both CRP and HOMA-IR levels, this variable was considered relevant for contextualizing the findings.

The results of the meta-analyses are presented in Figure 2, Figure 3, Figure 4, Figure 5, Figure 6, Figure 7, Figure 8, Figure 9, Figure 10, Figure 11, Figure 12, Figure 13, Figure 14, Figure 15, Figure 16, Figure 17, Figure 18 and Figure 19. In addition to the primary analyses, sensitivity analyses are presented for compounds in which studies were identified as outliers.

Eight entries from seven independent trials, comprising 308 participants, contributed to the pooled analysis of resveratrol supplementation on CRP (Figure 2). The random-effects model yielded a non-significant pooled mean difference (MD = −0.37 mg/L; 95% CI: −1.05 to 0.30; *p* = 0.223). Between-study heterogeneity was non-significant (Q(7) = 8.49, *p* = 0.292, τ = 0.46), suggesting reasonable consistency across trials despite the absence of a significant effect, although the wide prediction interval (−1.65 to 0.91 mg/L) crossed zero and indicated uncertainty in the expected effect across potential replications. Visual inspection of the residual funnel plot (Figure 3) showed slight leftward asymmetry, with all studies falling within the 95% pseudo-confidence region. Only 13.5% of the pooled weight came from trials at low risk of bias, and the certainty of the evidence was low (downgraded for risk of bias and imprecision).

Ten entries from nine independent trials, comprising 364 participants, contributed to the pooled analysis of resveratrol supplementation on HOMA-IR (Figure 4). The random-effects model likewise yielded a non-significant pooled mean difference (MD = −0.14; 95% CI: −0.35 to 0.08; *p* = 0.181), indicating no effect of resveratrol supplementation on insulin resistance. Between-study heterogeneity was low and non-significant (Q(9) = 7.25, *p* = 0.611, τ = 0.15), and the 95% prediction interval (−0.53 to 0.26) indicated consistent null findings across trials and made a clinically meaningful effect unlikely. Visual inspection of the residual funnel plot revealed no substantial asymmetry or evidence of publication bias (Figure 5). Only 1.5% of the pooled weight came from trials at low risk of bias, and the certainty of the evidence was moderate (downgraded for risk of bias).

**Figure 2:**
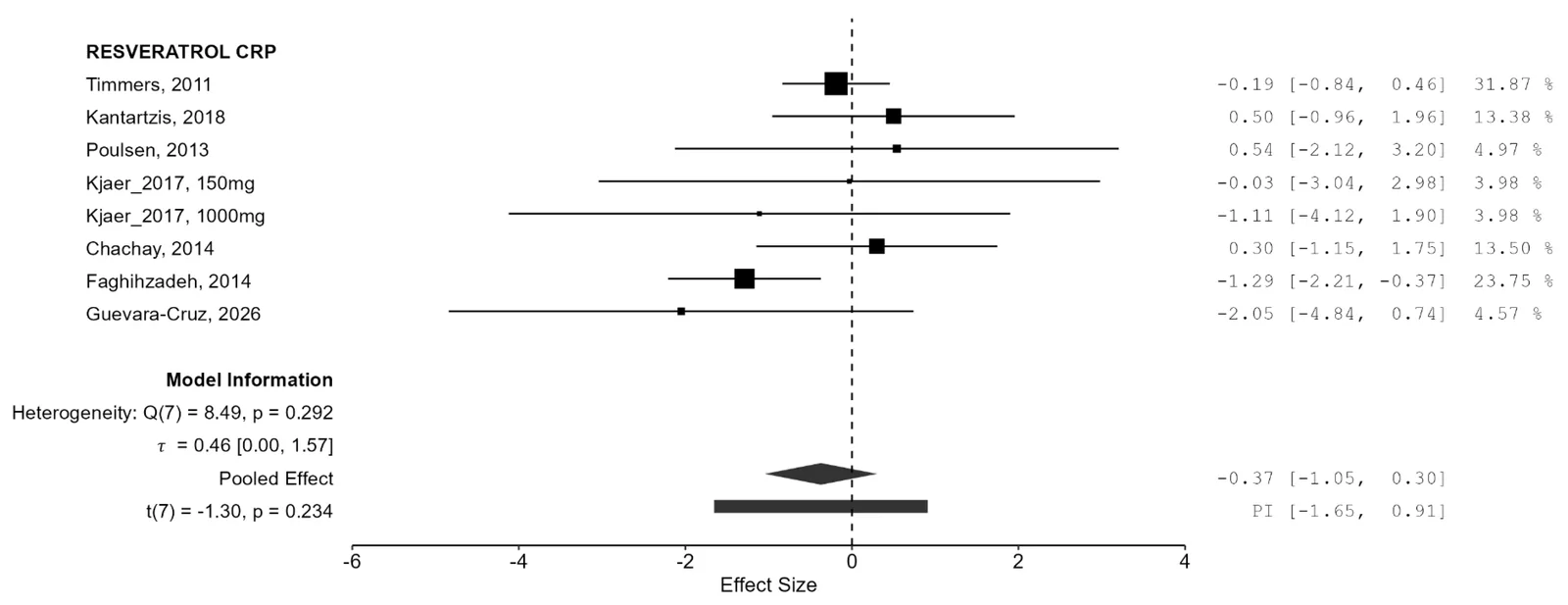
Effect of resveratrol supplementation on CRP (k = 8 entries from 7 independent trials; *n* = 308 distinct participants) [42,47,48,49,50,51,52]. Random-effects model, restricted maximum likelihood (REML) estimation of τ^2^ with Knapp–Hartung adjustment; effect size expressed as mean difference (mg/L). Kjær et al. (2017) [42] contributed two entries (150 and 1000 mg/day) sharing one placebo group, divided equally between them; Timmers et al. (2011) [47], a crossover trial, was analyzed with a paired-data adjustment (r = 0.5) Squares show study mean differences (size proportional to weight) with 95% CI (horizontal lines); the diamond shows the pooled effect and its 95% CI, and the bar below it the 95% PI. The dashed line indicates no effect. PI, prediction interval; τ, between-study standard deviation.

**Figure 3:**
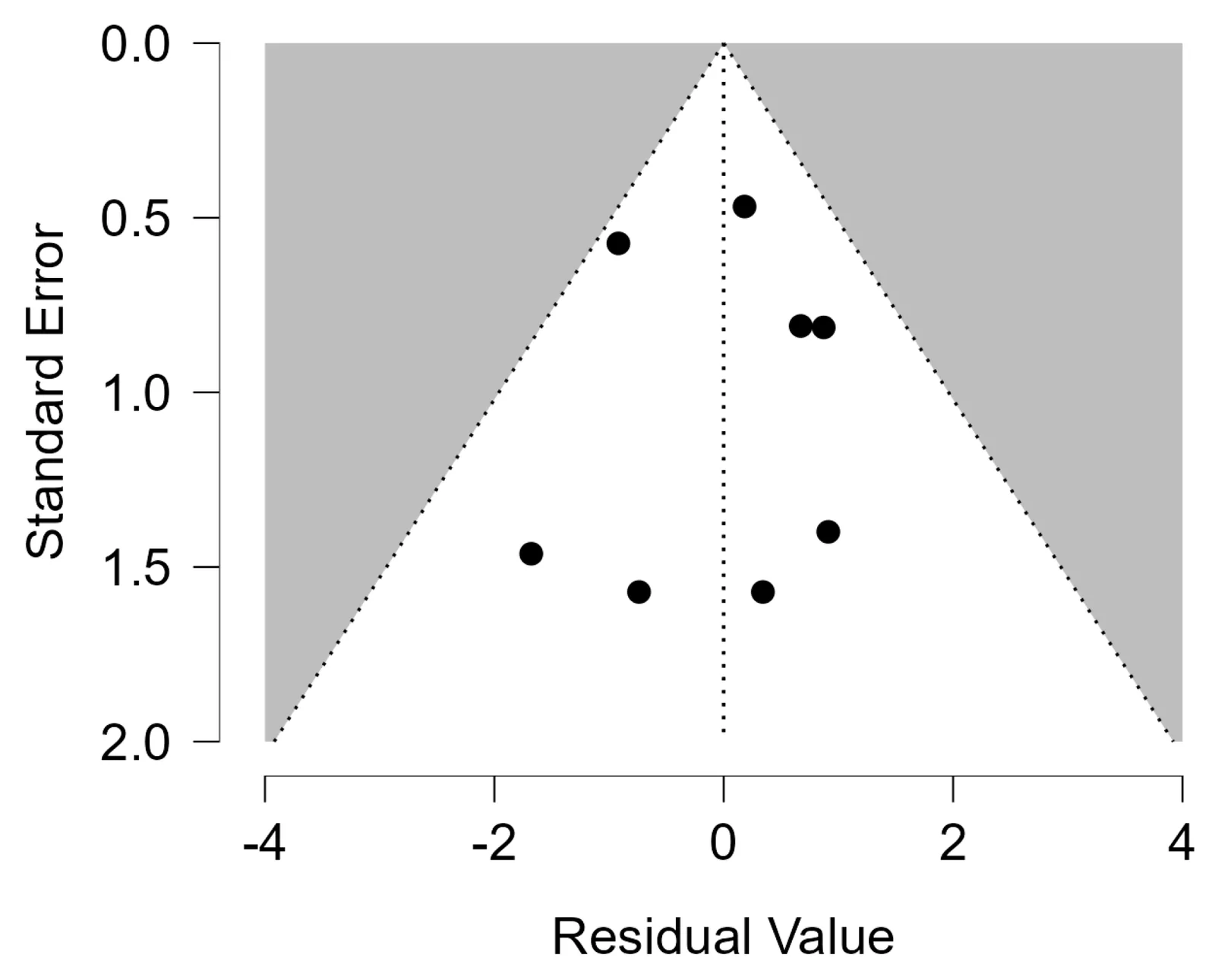
Residual funnel plot for the effect of resveratrol supplementation on CRP (k = 8), represented by the residual effect sizes against their standard errors. Each dot represents an individual study: the vertical dotted line indicates the model estimate, and the white triangular region represents the pseudo-95% confidence limits (±1.96 × SE). Points in the grey areas fall out-side these limits. All comparisons fell within the 95% pseudo-confidence region and no exclusions were made.

**Figure 4:**
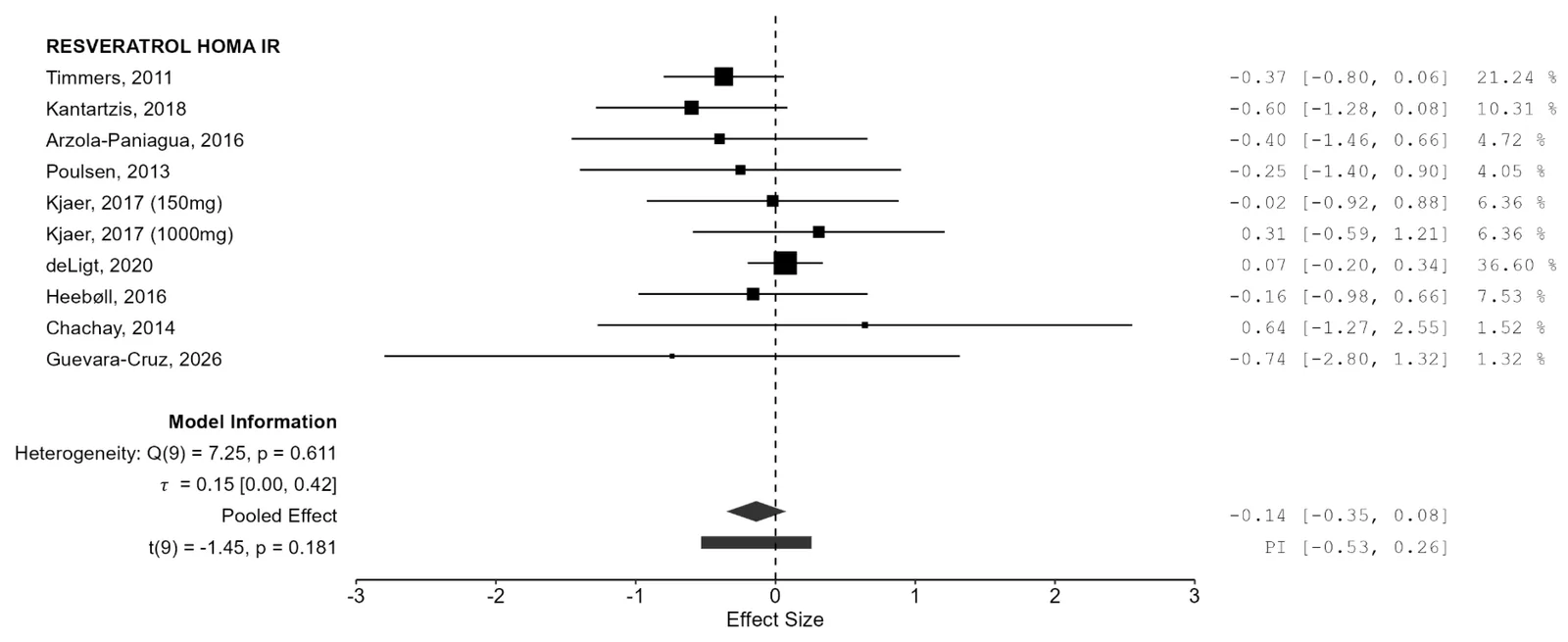
Effect of resveratrol supplementation on HOMA-IR (k = 10 entries from 9 independent trials; n = 364 distinct participants) [42,47,48,49,50,52,53,54,55]. Random-effects model, restricted maximum likelihood (REML) estimation of τ^2^ with Knapp–Hartung adjustment; effect size expressed as mean difference. Kjær et al. (2017) [42] contributed two entries (150 and 1000 mg/day) sharing one placebo group, divided equally between them; Timmers et al. (2011) [47], a crossover trial, was analyzed with a paired-data adjustment (r = 0.5). Squares show study mean differences (size proportional to weight) with 95% CI (horizontal lines); the diamond shows the pooled effect and its 95% CI, and the bar below it the 95% PI. The dashed line indicates no effect. PI, prediction interval; τ, between-study standard deviation.

**Figure 5:**
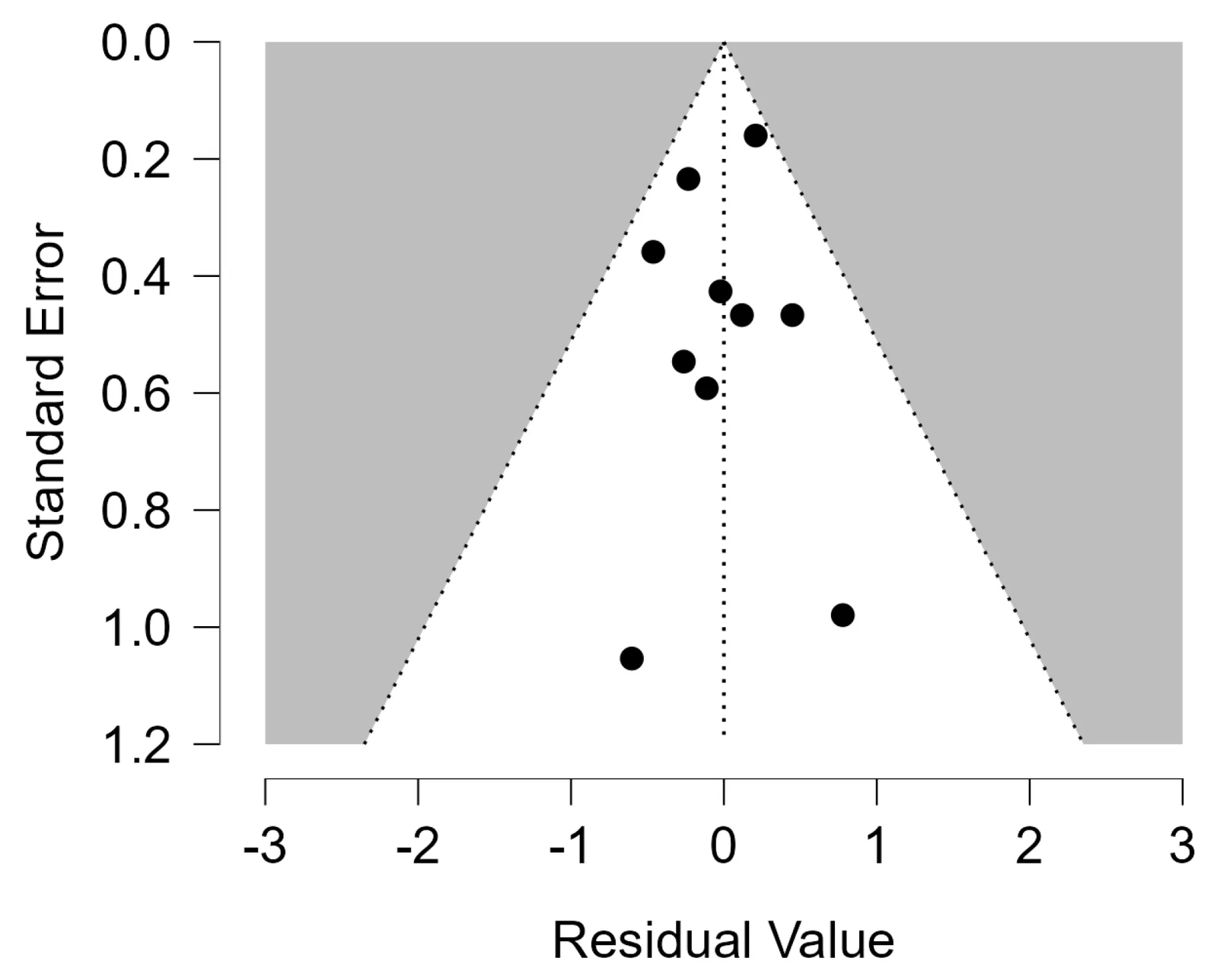
Residual funnel plot for the effect of resveratrol supplementation on HOMA-IR (k = 10), represented by the residual effect sizes against their standard errors. Each dot represents an individual study: the vertical dotted line indicates the model estimate, and the white triangular region represents the pseudo-95% confidence limits (±1.96 × SE). Points in the grey areas fall out-side these limits. All comparisons fell within the 95% pseudo-confidence region, with no substantial asymmetry observed, and no exclusions were made.

Eight entries, comprising 486 distinct participants, were initially included in the pooled analysis of curcumin supplementation on CRP (Figure 6). The random-effects model yielded a non-significant pooled mean difference (MD = −0.65 mg/L [−2.44, 1.14]; *p* = 0.417), with substantial heterogeneity (Q(7) = 32.74, *p* < 0.001, τ = 0.91) and a wide prediction interval (−3.45 to 2.15 mg/L). Visual inspection of the residual funnel plot (Figure 8) identified Amin et al. (2015) [48] as an outlier meeting the pre-established criterion for exclusion, with a markedly larger effect size than the remaining comparisons (MD = −14.00 mg/L [−20.66, −7.34]). Following its removal, the analysis was repeated on the remaining seven trials (n = 360 participants) (Figure 7). Heterogeneity decreased (Q(6) = 16.48, *p* = 0.011, τ = 0.67) but the pooled effect remained non-significant (MD = −0.44 mg/L [−1.44, 0.55]; *p* = 0.315), with a prediction interval of −2.35 to 1.46 mg/L, and no further outliers were identified. These findings indicate that the effect of curcumin on systemic inflammation remains uncertain irrespective of the inclusion of Amin et al. (2015). A total of 28.7% of the pooled weight came from trials at low risk of bias (24.3% at high risk), and the certainty of the evidence was very low (downgraded for risk of bias, inconsistency and imprecision).

Seven trials, comprising 366 participants, contributed to the pooled analysis of curcumin supplementation on HOMA-IR (Figure 9). Campbell et al. (2019) [41] was excluded prior to analysis by consensus among the authors because the final fasting insulin in the curcumin group (41.71 ± 19.36 mIU/L) was about six times that of the placebo group (7.64 ± 0.60 mIU/L), yielding a derived HOMA-IR of 9.86 ± 14.54 (versus 1.77 ± 0.48), a value incompatible with non-diabetic adults. In contrast to CRP, the random-effects model yielded a statistically significant pooled mean difference favoring the intervention (MD = −0.30 [−0.53, −0.07]; *p* = 0.019). Between-study heterogeneity was low and non-significant (Q(6) = 7.53, *p* = 0.275, τ = 0.13), suggesting consistency across trials, although the prediction interval crossed zero (−0.70 to 0.10), indicating uncertainty in the expected effect across potential replications despite the significant pooled estimate. Visual inspection of the residual funnel plot (Figure 10) revealed Cicero et al. (2020) [53] as a borderline case; as it did not meet the criterion for exclusion, it was retained in the final analysis. Most of the pooled weight (72.3%) came from trials at low risk of bias and none from trials at high risk; the certainty of the evidence was moderate (downgraded for imprecision).

**Figure 6:**
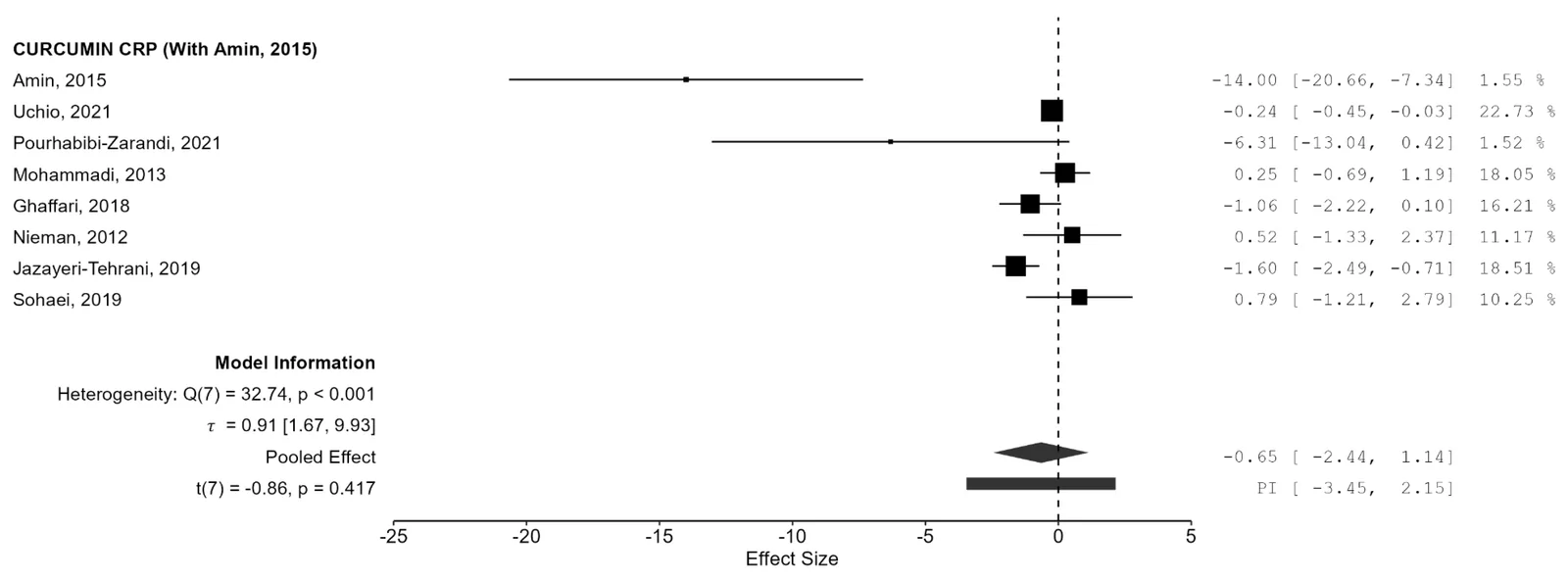
Effect of curcumin supplementation on CRP (k = 8 trials; n = 486 participants) [56,57,58,59,60,61,62,63]. Random-effects model, REML estimation of τ^2^ with Knapp–Hartung adjustment; effect size expressed as mean difference (mg/L). Mohammadi et al. (2013) [49] and Nieman et al. (2012) [54], crossover trials, were analyzed with a paired-data adjustment (r = 0.5). Squares show study mean differences (size proportional to weight) with 95% CI (horizontal lines); the diamond shows the pooled effect and its 95% CI, and the bar below it the 95% PI. The dashed line indicates no effect. PI, prediction interval; τ, between-study standard deviation.

**Figure 7:**
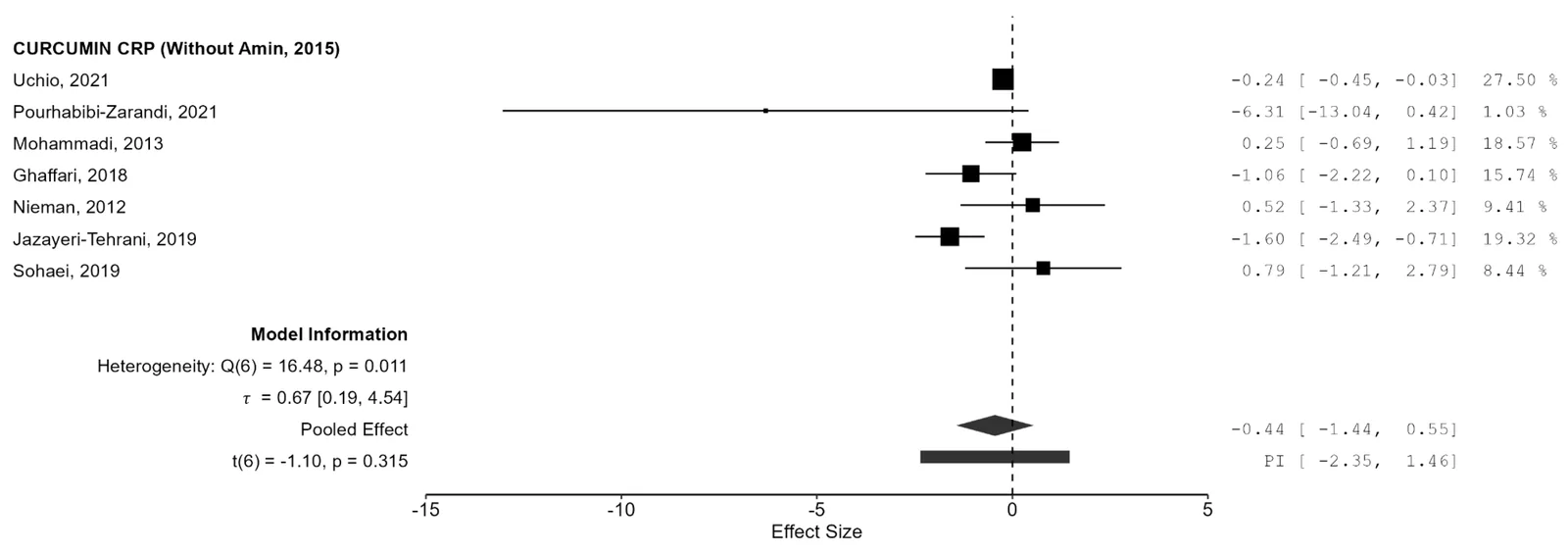
Effect of curcumin supplementation on CRP, sensitivity analysis after exclusion of outlier Amin et al. (2015) (k = 7 trials; n = 360 participants) [57,58,59,60,61,62,63]. Random-effects model, REML estimation of τ^2^ with Knapp–Hartung adjustment; effect size expressed as mean difference (mg/L). Mohammadi et al. (2013) and Nieman et al. (2012), crossover trials, were analyzed with a paired-data adjustment (r = 0.5). Squares show study mean differences (size proportional to weight) with 95% CI (horizontal lines); the diamond shows the pooled effect and its 95% CI, and the bar below it the 95% PI. The dashed line indicates no effect. PI, prediction interval; τ, between-study standard deviation.

**Figure 8:**
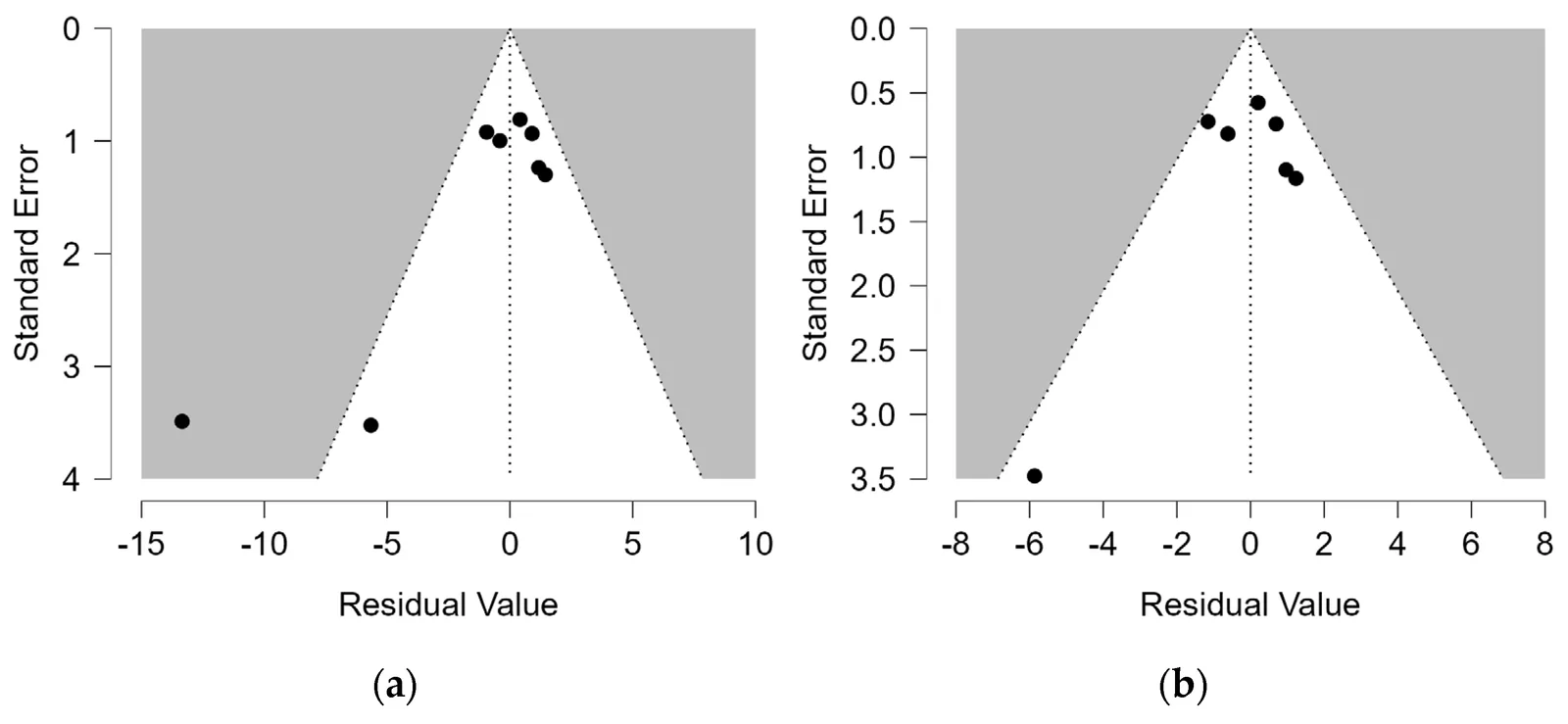
Residual funnel plots for the effect of curcumin supplementation on CRP, represented by the residual effect sizes against their standard errors. Each dot represents an individual study: the vertical dotted line indicates the model estimate, and the white triangular region represents the pseudo-95% confidence limits (±1.96 × SE). Points in the grey areas fall out-side these limits (**a**) primary analysis of all included studies (k = 8); (**b**) excluding identified outlier Amin et al. (2015) (k = 7) [48]. All remaining comparisons fell within the 95% pseudo-confident region.

**Figure 9:**
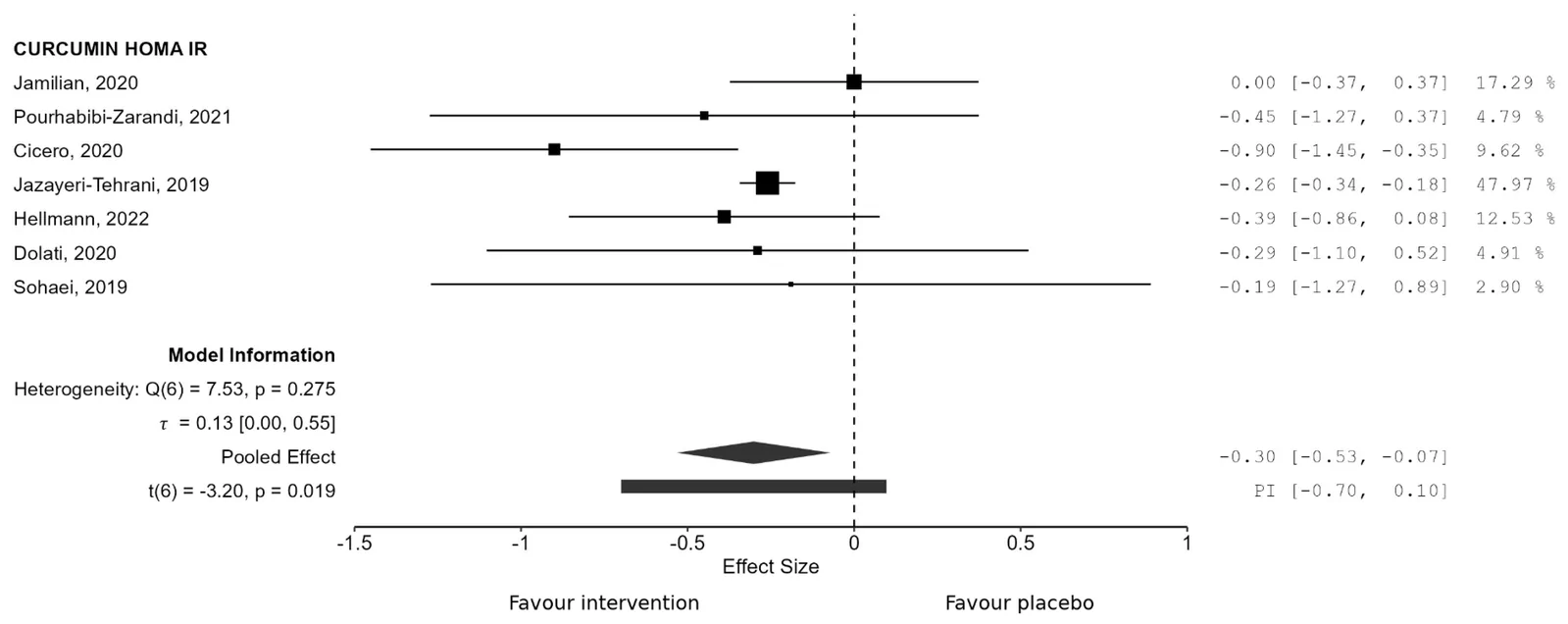
Effect of curcumin supplementation on HOMA-IR (k = 7 trials; n = 366 participants) [58,62,63,64,65,66,67]. Random-effects model, REML estimation of τ^2^ with Knapp–Hartung adjustment; effect size expressed as mean difference in HOMA-IR units. Hellmann et al. (2022) [55] reported HOMA2-IR rather than classical HOMA-IR. Squares show study mean differences (size proportional to weight) with 95% CI (horizontal lines); the diamond shows the pooled effect and its 95% CI, and the bar below it the 95% PI. The dashed line indicates no effect. PI, prediction interval; τ, between-study standard deviation.

**Figure 10:**
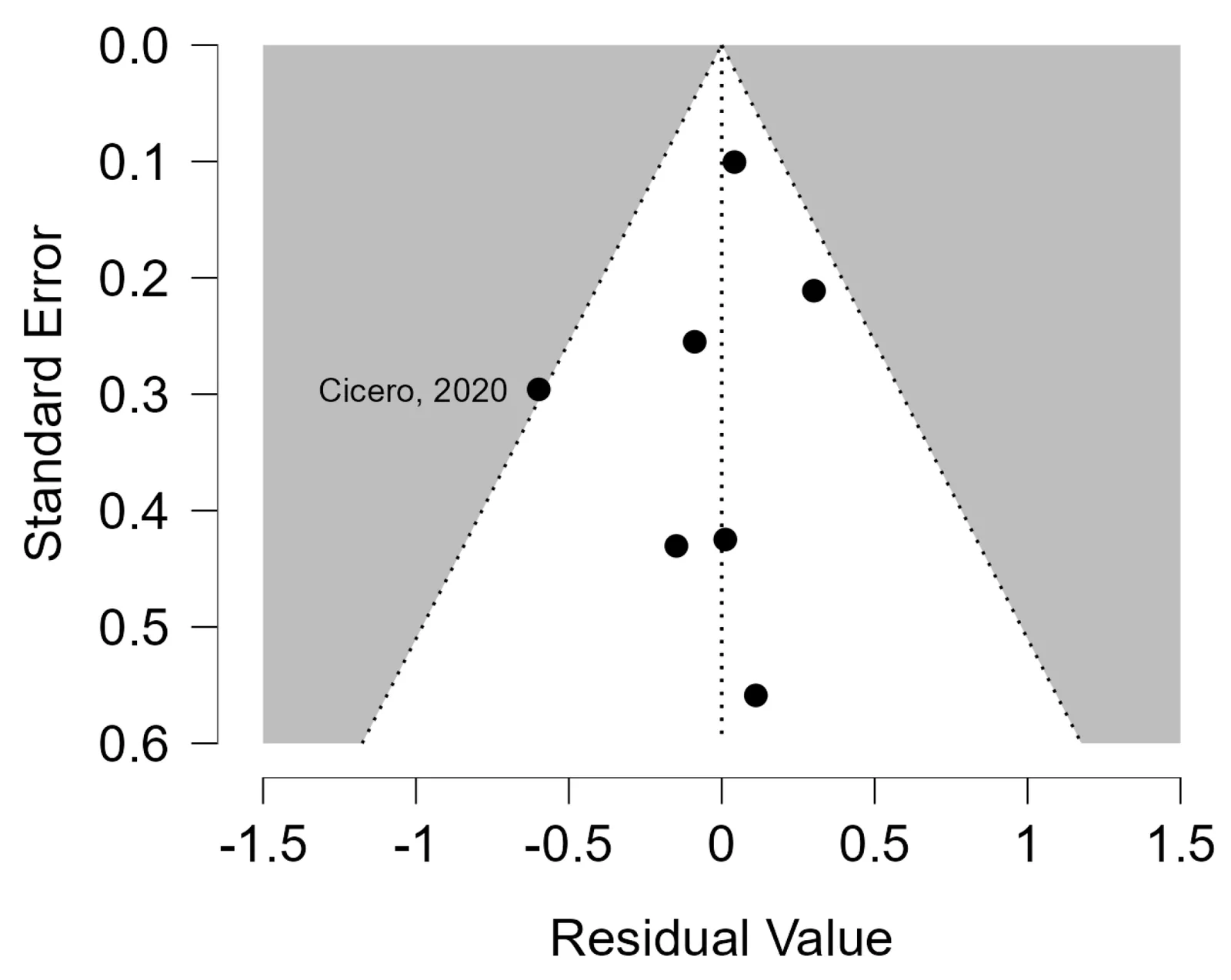
Residual funnel plot for the effect of curcumin supplementation on HOMA-IR (k = 7), represented by the residual effect sizes against their standard errors. Each dot represents an individual study: the vertical dotted line indicates the model estimate, and the white triangular region represents the pseudo-95% confidence limits (±1.96 × SE). Points in the grey areas fall out-side these limits. Cicero et al. (2020) [53] lay near the boundary of the 95% pseudo-confidence region but did not fall entirely outside it and was retained; all remaining comparisons fell within the region and no exclusions were made.

Seven trials, comprising 409 distinct participants, contributed to the pooled analysis of green tea supplementation on CRP (Figure 11). The random-effects model yielded a borderline pooled mean difference falling at the threshold of conventional statistical significance (MD = −0.43 mg/L [−0.87, 0.02]; *p* = 0.057), with a confidence interval nearly excluding zero. Between-study heterogeneity was substantial and significant (Q(6) = 40.41, *p* < 0.001, τ = 0.42), and the prediction interval crossed zero (−1.55 to 0.69 mg/L), indicating uncertainty in the expected effect across potential replications. Visual inspection of the residual funnel plot (Figure 12) revealed no substantial asymmetry; no exclusions were made. Although the pooled estimate does not reach conventional significance at α = 0.05, the near-significant *p*-value and the confidence interval approaching zero suggest a possible trend towards CRP reduction; however, given the substantial heterogeneity, the results should be interpreted with caution. Trials at low risk of bias contributed 53.1% of the pooled weight and none was at high risk; the certainty of the evidence was low (downgraded for inconsistency and imprecision).

Ten trials, comprising 798 participants, contributed to the pooled analysis of green tea supplementation on HOMA-IR (Figure 13). The random-effects model yielded a non-significant pooled mean difference (MD = −0.15 [−0.63, 0.33]; *p* = 0.508), with substantial and significant heterogeneity (Q(9) = 31, *p* < 0.001, τ = 0.53). Visual inspection of the residual funnel plot (Figure 15) identified Hussain et al. (2017) [50] as an outlier meeting the pre-established criterion for exclusion; upon its removal, Bogdanski et al. (2012) [51] emerged as a second outlier and was likewise excluded. Following the sequential exclusion of both trials, the analysis was repeated on the remaining eight trials (n = 662 participants) (Figure 14), and no further outliers were identified. Heterogeneity was rendered negligible and non-significant (Q(7) = 5.03, *p* = 0.656, τ = 0.00) and the pooled effect remained non-significant (MD = 0.10 [−0.07, 0.27]; *p* = 0.20), with an identical prediction interval (−0.07 to 0.27), indicating a robust and consistent null finding (Figure 14). We consider that the reversal in the direction of the estimate towards placebo further reinforces the absence of a meaningful effect of green tea supplementation on insulin resistance. In the primary analysis of ten trials, 46.5% of the pooled weight came from trials at low risk of bias and 5.0% from trials at high risk, but the certainty of the evidence was very low (downgraded for risk of bias, inconsistency and imprecision), largely owing to the two trials identified as outliers: in the sensitivity analysis restricted to the remaining eight trials, 56.1% of the weight came from trials at low risk of bias, and the certainty of the evidence would be high.

**Figure 11:**
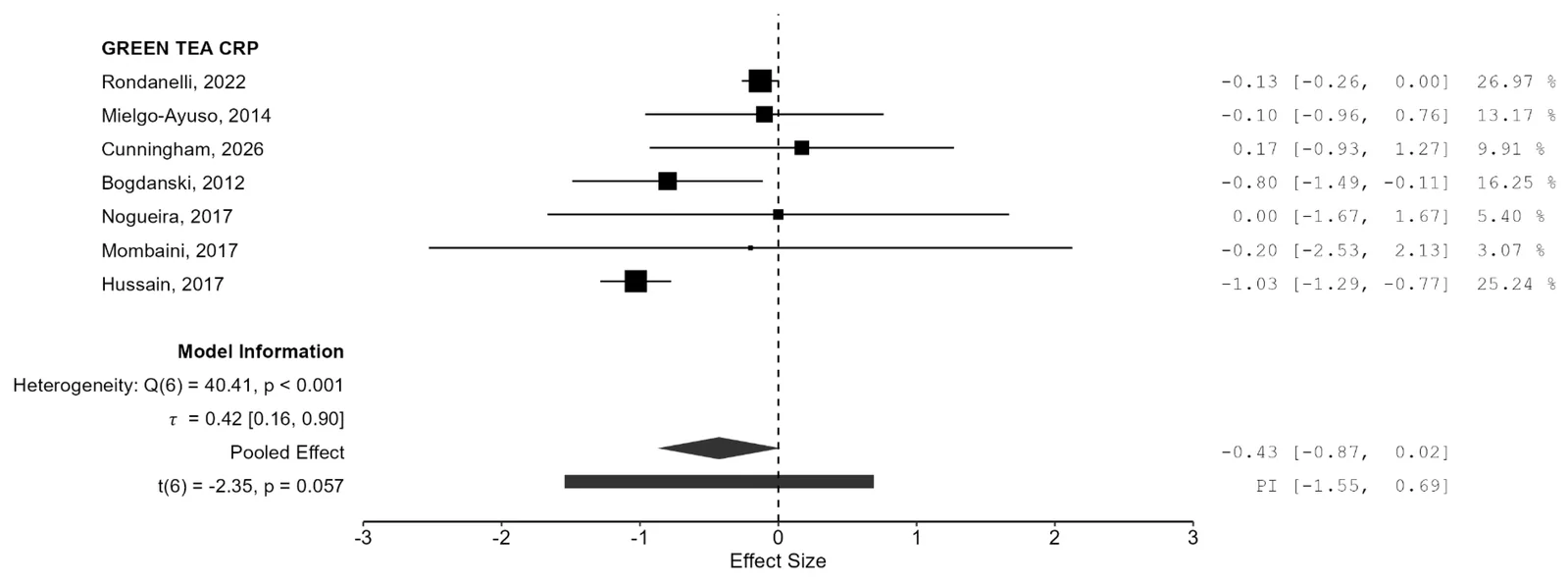
Effect of green tea supplementation on CRP (k = 7 trials; n = 409 participants) [50,51,52,56,57,58,64]. Random-effects model, REML estimation of τ^2^ with Knapp–Hartung adjustment; effect size expressed as mean difference (mg/L). Nogueira et al. (2017) [64], a crossover trial. Squares show study mean differences (size proportional to weight) with 95% CI (horizontal lines); the diamond shows the pooled effect and its 95% CI, and the bar below it the 95% PI. The dashed line indicates no effect. PI, prediction interval; τ, between-study standard deviation.

**Figure 12:**
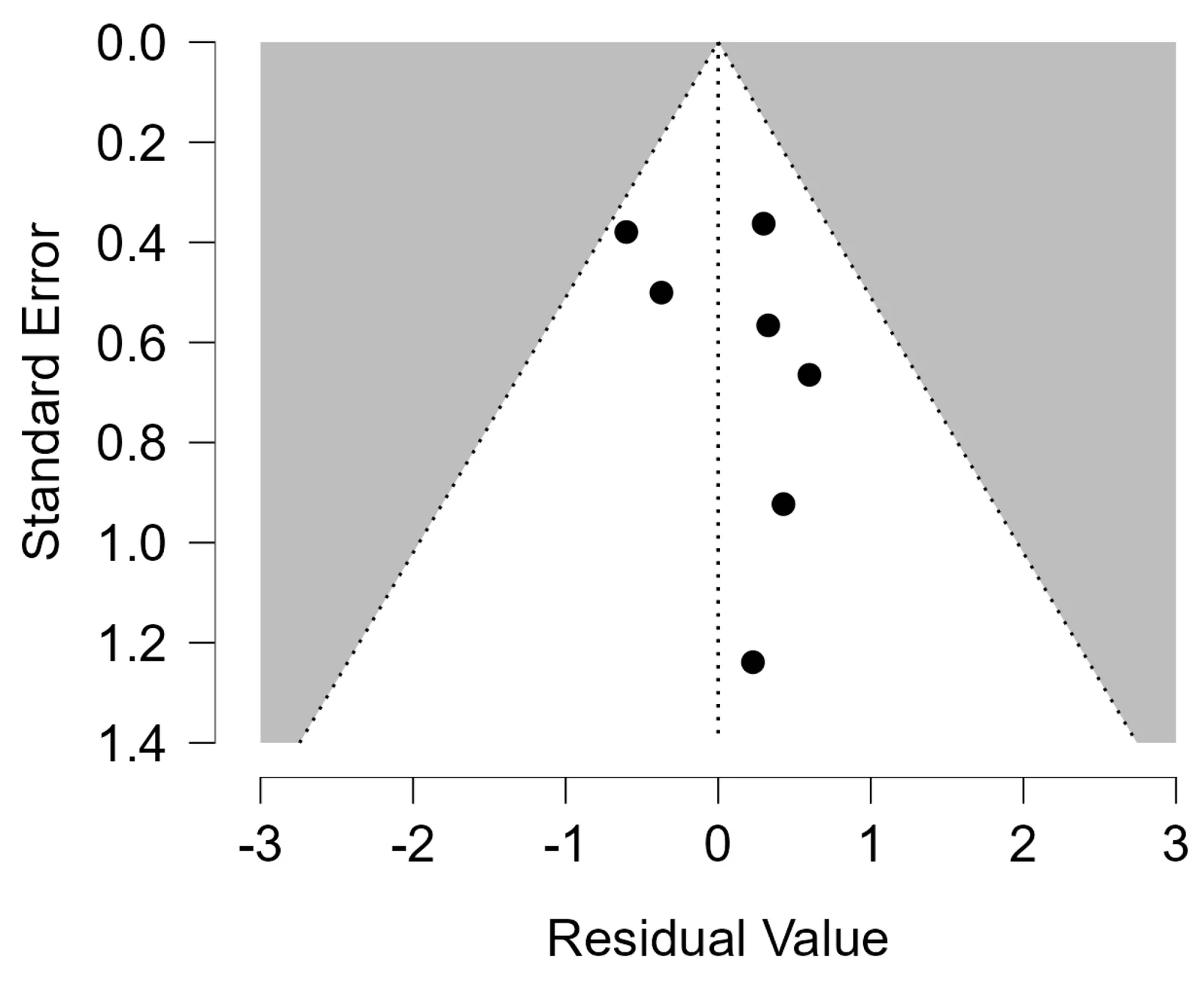
Residual funnel plot for the effect of green tea supplementation on CRP (k = 7), represented by the residual effect sizes against their standard errors. Each dot represents an individual study: the vertical dotted line indicates the model estimate, and the white triangular region represents the pseudo-95% confidence limits (±1.96 × SE). Points in the grey areas fall out-side these limits. All comparisons fell within the 95% pseudo-confidence region and no exclusions were made.

**Figure 13:**
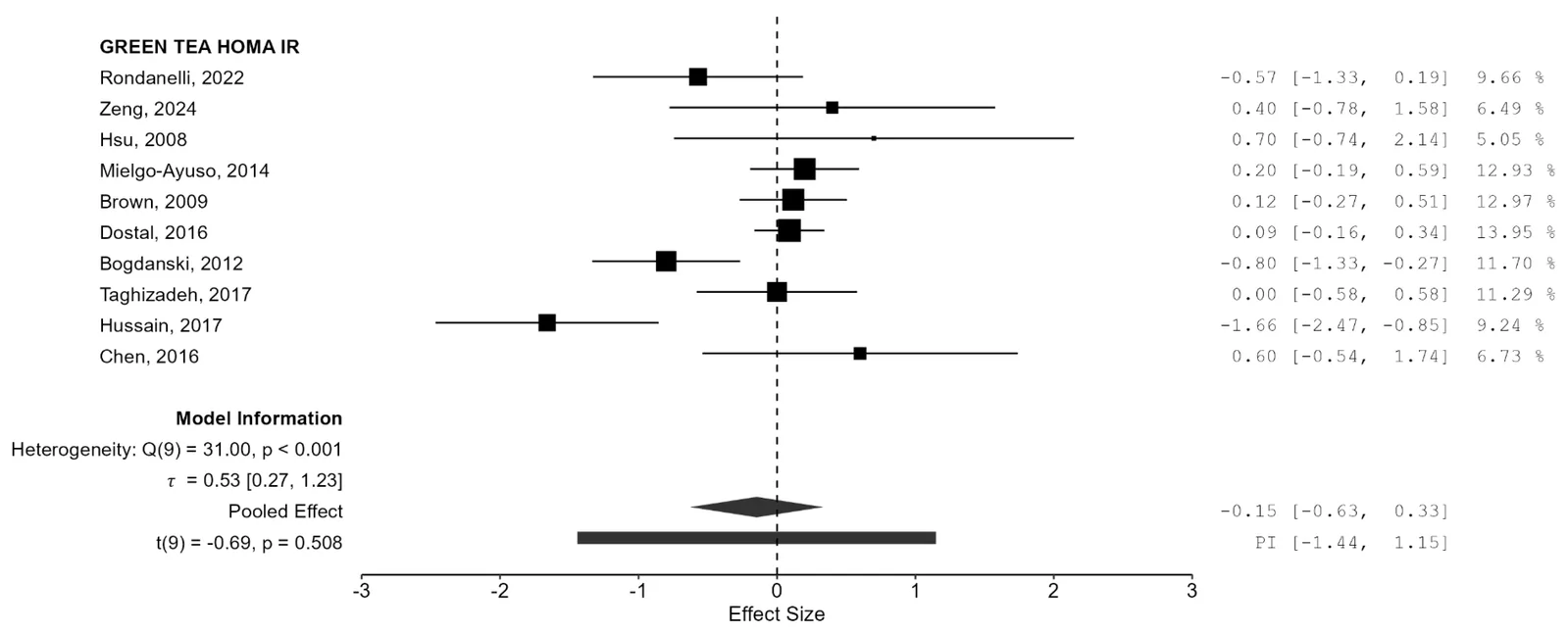
Effect of green tea supplementation on HOMA-IR (k = 10 trials; n = 798 participants) [68,69,70,71,72,73,74,75,76,77]. Random-effects model, REML estimation of τ^2^ with Knapp–Hartung adjustment; effect size expressed as mean difference in HOMA-IR units. Zeng et al. (2024) [65], a crossover trial, was analyzed with a paired-data adjustment (r = 0.5); Taghizadeh et al. (2017) [59] administered green tea in combination with capsaicin and ginger, retained following a protocol-level exception.; Squares show study mean differences (size proportional to weight) with 95% CI (horizontal lines); the diamond shows the pooled effect and its 95% CI, and the bar below it the 95% PI. The dashed line indicates no effect. PI, prediction interval; τ, between-study standard deviation.

**Figure 14:**
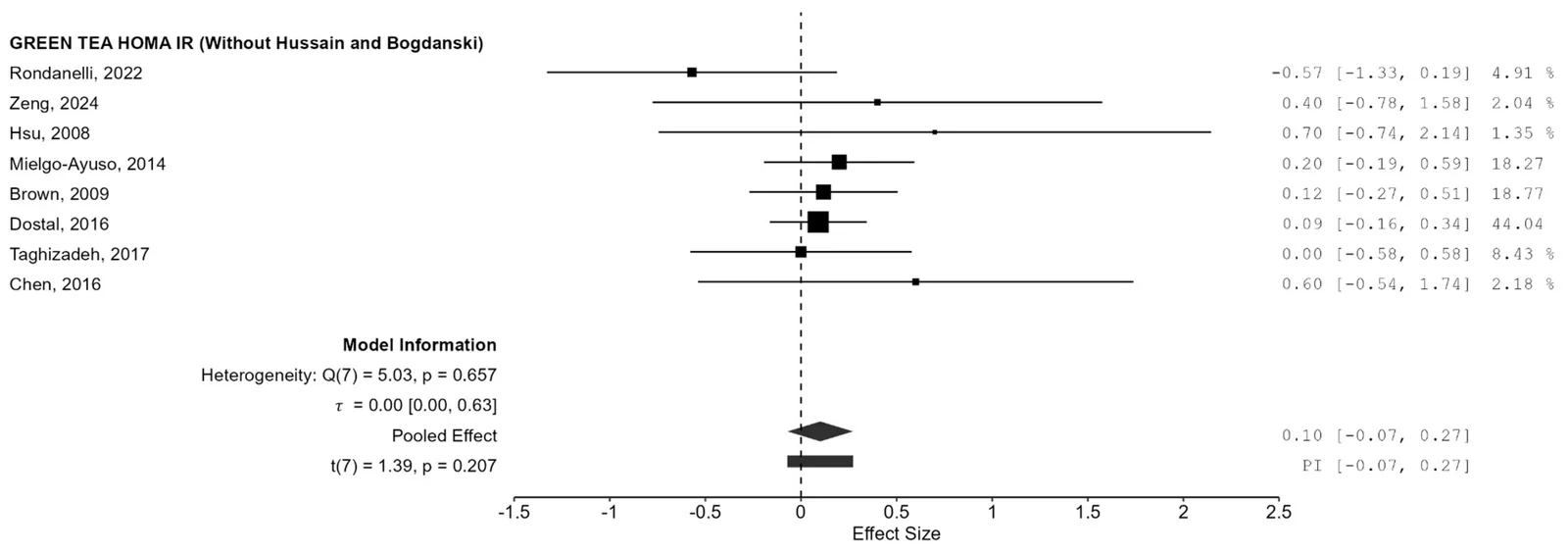
Sensitivity analysis of the effect of green tea supplementation on HOMA-IR after sequential exclusion of identified outliers Hussain et al. (2017) [50] and Bogdanski et al. (2012) [51] (k = 8 trials; n = 662 participants) [68,69,70,71,72,73,75,77]. Random-effects model, REML estimation of τ^2^ with Knapp–Hartung adjustment; effect size expressed as mean difference in HOMA-IR units. Zeng et al. (2024), a crossover trial, was analyzed with a paired-data adjustment (r = 0.5); Taghizadeh et al. (2017) administered green tea in combination with capsaicin and ginger, retained following a protocol-level exception. Squares show study mean differences (size proportional to weight) with 95% CI (horizontal lines); the diamond shows the pooled effect and its 95% CI, and the bar below it the 95% PI. The dashed line indicates no effect. PI, prediction interval; τ, between-study standard deviation.

**Figure 15:**
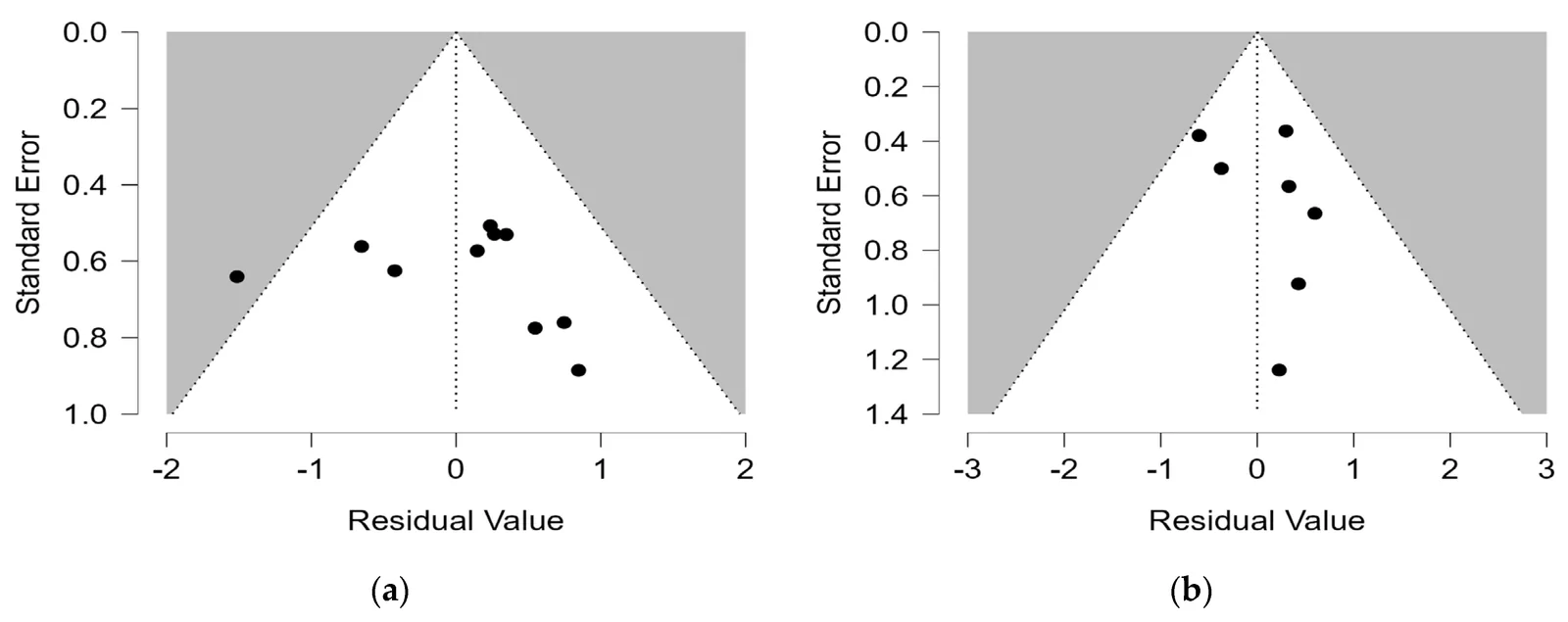
Residual funnel plots for the effect of green tea supplementation on HOMA-IR, represented by the residual effect sizes against their standard errors. Each dot represents an individual study: the vertical dotted line indicates the model estimate, and the white triangular region represents the pseudo-95% confidence limits (±1.96 × SE). Points in the grey areas fall out-side these limits. (**a**) primary analysis (k = 10); (**b**) sensitivity analysis excluding identified outliers Hussain et al. (2017) [50] and Bogdanski et al. (2012) [51] (k = 8). Both trials fell outside the 95% pseudo-confidence region and were removed sequentially, Bogdanski et al. (2012) [51] having emerged as an outlier only after the removal of Hussain et al. (2017) [50]; all remaining comparisons fell within the region.

Fifteen trials, comprising 767 distinct participants, contributed to the pooled analysis of omega-3 supplementation on CRP (Figure 16). The random-effects model yielded a non-significant pooled mean difference (MD = −0.16 mg/L [−0.42, 0.11]; *p* = 0.227), with substantial and significant heterogeneity (Q(14) = 50.91, *p* < 0.001, τ = 0.28) and a wide prediction interval (−0.81 to 0.50 mg/L), indicating uncertainty in the expected effect across potential replications. Visual inspection of the residual funnel plot (Figure 17) suggested mild asymmetry towards larger reductions in the intervention arm, alongside comparisons lying near the boundary of the 95% pseudo-confidence region; as none met the pre-established criterion for exclusion, no exclusions were made. Taken together, the non-significant pooled estimate, the substantial heterogeneity and the mildly asymmetric funnel plot indicate that the effect of omega-3 supplementation on systemic inflammation remains inconclusive within this dataset. None of the pooled weight came from trials at low risk of bias and 10.2% came from trials at high risk, and the certainty of the evidence was low (downgraded for risk of bias and inconsistency).

Twenty-one entries from nineteen independent trials, comprising 1008 distinct participants, contributed to the pooled analysis of omega-3 supplementation on HOMA-IR (Figure 18). The random-effects model likewise yielded a non-significant pooled mean difference (MD = −0.04 [−0.21, 0.14]; *p* = 0.672), the near-zero pooled estimate indicating not only statistical non-significance but also a negligible magnitude of effect. Between-study heterogeneity was substantial and significant (Q(20) = 47.60, *p* < 0.001, τ = 0.29), and the wide prediction interval spanned both directions similarly (−0.66 to 0.59), indicating considerable variability in the expected effect across potential replications. Visual inspection of the residual funnel plot (Figure 19) revealed one comparison as a borderline case; as it did not meet the criterion for exclusion, it was retained and the pooled result interpreted with caution. Trials at low risk of bias contributed 16.1% of the pooled weight and 11.5% came from trials at high risk; the certainty of the evidence was low (downgraded for risk of bias and inconsistency).

**Figure 16:**
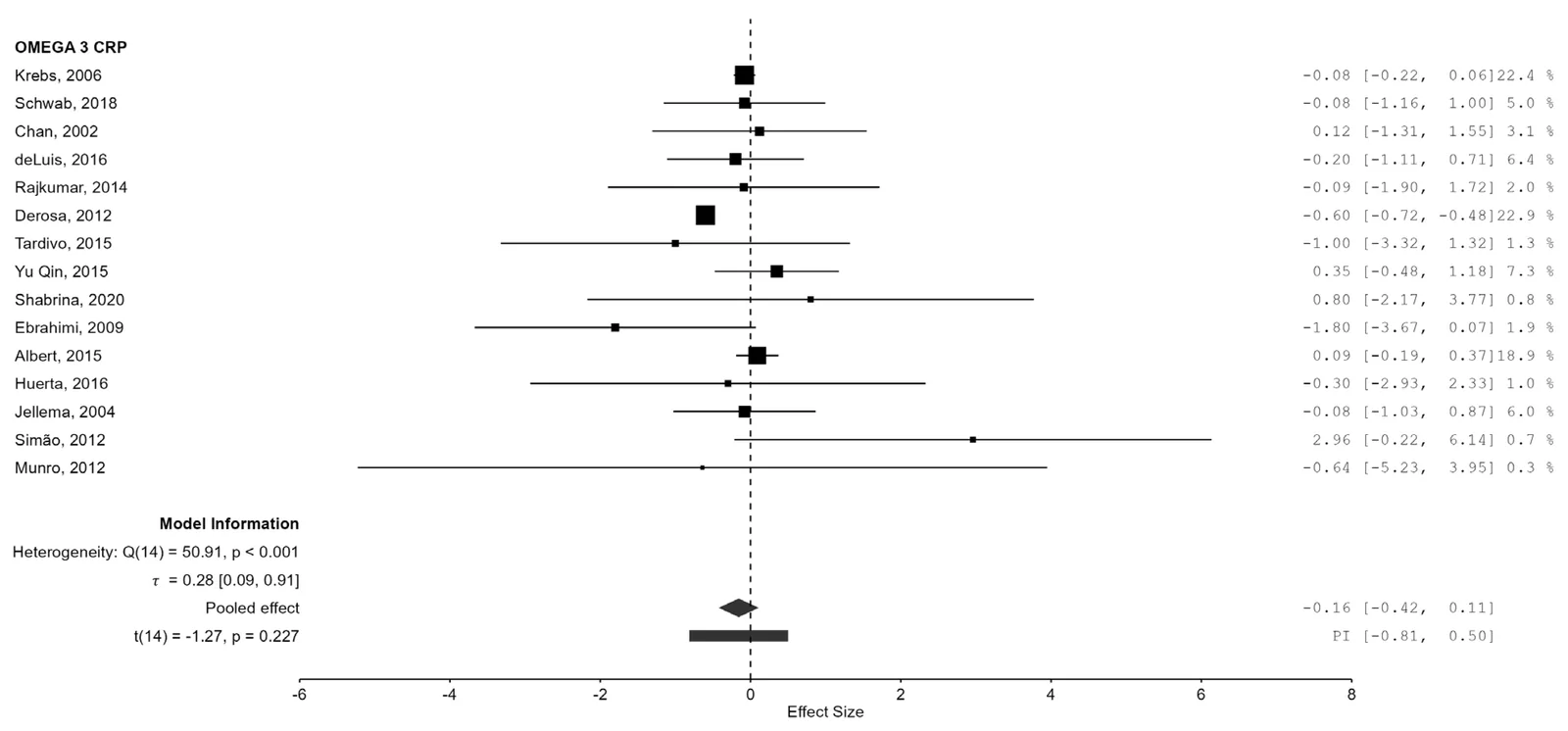
Effect of omega-3 supplementation on CRP (k = 15 trials; n = 767 participants) [78,79,80,81,82,83,84,85,86,87,88,89,90,91,92]. Random-effects model, REML estimation of τ^2^ with Knapp–Hartung adjustment; effect size expressed as mean difference (mg/L). Control conditions varied across trials, comprising placebo capsules, controlled diet or usual diet without supplementation. Albert et al. (2015) [60] and Jellema et al. (2004) [61], crossover trials, were analyzed with a paired-data adjustment (r = 0.5). Squares show study mean differences (size proportional to weight) with 95% CI (horizontal lines); the diamond shows the pooled effect and its 95% CI, and the bar below it the 95% PI. The dashed line indicates no effect. PI, prediction interval; τ, between-study standard deviation.

**Figure 17:**
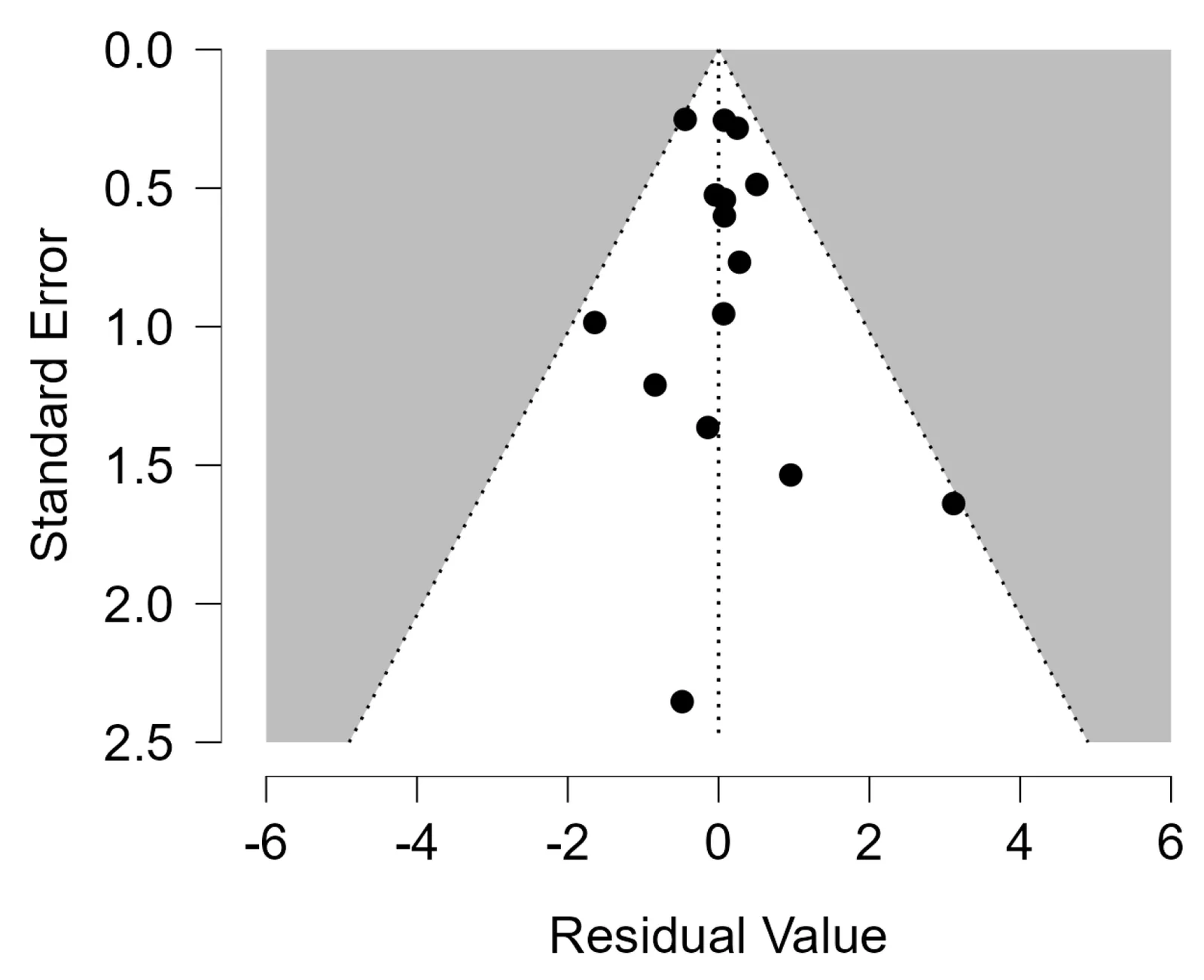
Residual funnel plot for the effect of omega-3 supplementation on CRP (k = 15), represented by the residual effect sizes against their standard errors. Each dot represents an individual study: the vertical dotted line indicates the model estimate, and the white triangular region represents the pseudo-95% confidence limits (±1.96 × SE). Points in the grey areas fall out-side these limits. One comparison lay near the boundary of the 95% pseudo-confidence region but did not fall entirely outside it and was retained. Mild asymmetry was observed towards larger reductions in the intervention arm; all comparisons fell within the 95% pseudo-confidence region and no exclusions were made.

**Figure 18:**
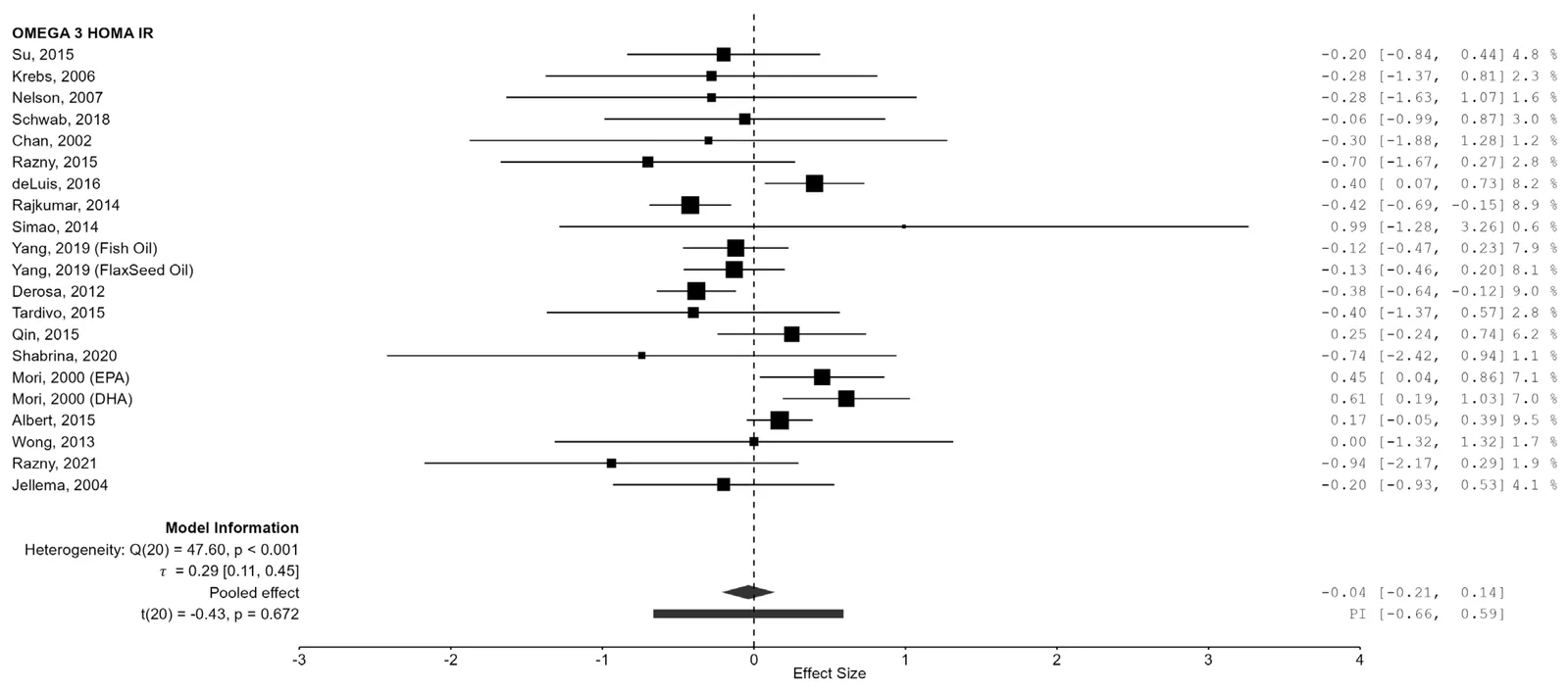
Effect of omega-3 supplementation on HOMA-IR (k = 21 entries from 19 independent trials; n = 1008 participants) [43,44,78,80,82,83,84,85,86,87,88,90,92,93,94,95,96,97,98]. Random-effects model, REML estimation of τ^2^ with Knapp–Hartung adjustment; effect size expressed as mean difference in HOMA-IR units. Control conditions varied across trials, comprising placebo capsules, controlled diet or usual diet without supplementation. Mori et al. (2000) (EPA and DHA) [43] and Yang et al. (2019) (fish oil and flaxseed oil) [44] each contributed two entries sharing one control group, divided equally between them; Albert et al. (2015) [60] and Jellema et al. (2004) [61], crossover trials, were analyzed with a paired-data adjustment (r = 0.5).; Squares show study mean differences (size proportional to weight) with 95% CI (horizontal lines); the diamond shows the pooled effect and its 95% CI, and the bar below it the 95% PI. The dashed line indicates no effect. PI, prediction interval; τ, between-study standard deviation.

**Figure 19:**
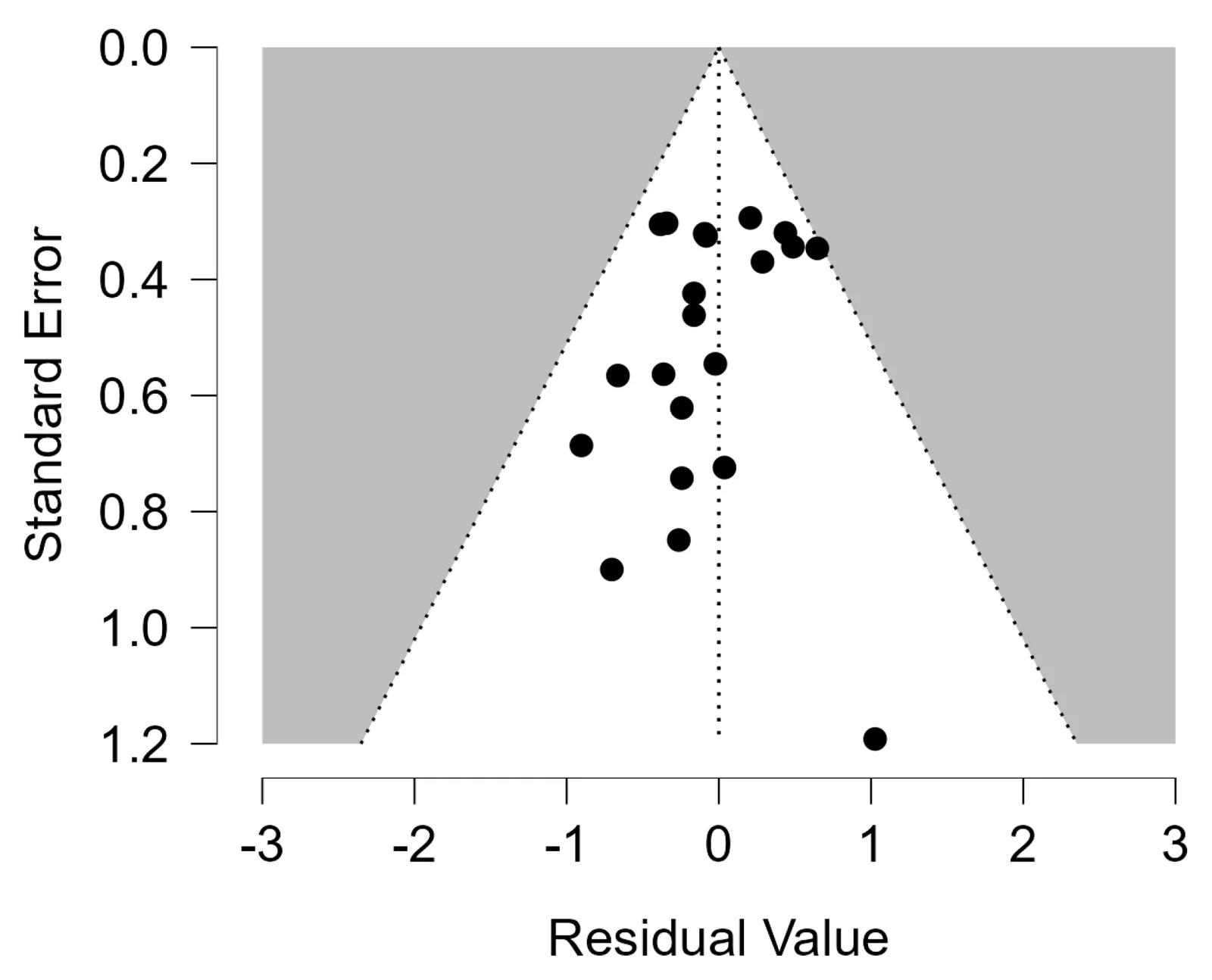
Residual funnel plot for the effect of omega-3 supplementation on HOMA-IR (k = 21), represented by the residual effect sizes against their standard errorEach dot represents an individual study: the vertical dotted line indicates the model estimate, and the white triangular region represents the pseudo-95% confidence limits (±1.96 × SE). Points in the grey areas fall outside these limits. All fcomparisons fell within the region and no exclusions were made.

The certainty of the evidence for each outcome is summarized in Table 3, Table 4, Table 5 and Table 6, organized per compound. The certainty ranged from very low to moderate, and none of the eight outcomes reached high certainty. Full evidence profiles, with the judgment for each GRADE domain, are provided in Supplementary File S4. The risk-of-bias assessment of each trial is provided in Supplementary Files S2 and S3.

## 4. Discussion

Across the eight independent pooled analyses conducted, only one yielded a statistically significant result: curcumin supplementation was associated with a significant reduction in HOMA-IR, with a moderate certainty of evidence. The remaining seven analyses failed to demonstrate significant effects at the conventional 95% confidence level, with green tea extract showing a borderline result for CRP that narrowly missed statistical significance. Additionally, the certainty of the evidence was low or very low for six of the eight outcomes and moderate for two (resveratrol and curcumin on HOMA-IR); none reached high certainty.

The limitations of translating promising pre-clinical findings into robust clinical outcomes are well established in the bioactive compound literature, and the present results are no exception. This translation remains particularly challenging in the heterogeneous context of overweight and obesity. Bioactive compounds have more frequently been evaluated in populations defined by other inflammatory or metabolic disorders that co-occur with excess adiposity, such as metabolic syndrome, NAFLD, or type 2 diabetes, making it difficult to isolate the effect attributable to overweight or obesity per se.

Prior meta-analyses have addressed related questions across different populations and outcomes; while some of the present findings align with this prior evidence, others diverge from it. Regarding omega-3 fatty acids, Sadeghi et al. (2017) [26] evaluated omega-3 supplementation on insulin resistance in women with polycystic ovary syndrome and found no significant effect on HOMA-IR, consistent with our findings, as did Amlashi et al. (2025) [34], whose dose–response meta-analysis did not demonstrate significant CRP reductions in overweight and obese participants. For green tea, Liu et al. (2013) [27] reported significant reductions in fasting glucose and HbA1c across seventeen trials, although these outcomes differ from the primary endpoints examined here; more directly comparable is Dehzad et al. (2025) [35], who likewise found that green tea failed to significantly reduce CRP at the 95% confidence level, mirroring our borderline result and reinforcing the interpretation that any anti-inflammatory effect, if present, is modest and insufficiently consistent. In the case of resveratrol, prior meta-analyses have reported significant CRP reductions in populations with metabolic syndrome and other inflammatory disorders [28,31]; no meta-analysis specifically targeting overweight or obese populations was identified, and the null findings reported here suggest that effects observed in more clinically severe populations may not generalize to individuals characterized primarily by excess adiposity. Regarding curcumin, the significant effect on HOMA-IR observed here is consistent with prior evidence in metabolic syndrome populations [29,33]; as no prior meta-analysis specifically targeting overweight and obese populations was identified for this outcome, the present study represents a novel contribution to this evidence gap. For CRP, Dehzad et al. (2023) [32] reported significant curcumin-associated reductions in obesity subgroup analyses, a finding that the present analysis could not replicate at conventional significance, likely reflecting differences in inclusion criteria, number of studies, and analytical approach. At the broader dietary pattern level, Poulsen, Lambert, and Jeppesen (2020) [30] demonstrated significant overall CRP reductions with diets high in plant-based products across subgroups including individuals with overweight and obesity, suggesting that isolated supplementation may be insufficient to produce the magnitude of anti-inflammatory effect achievable through comprehensive dietary modification.

The relationship between dose, formulation, and outcome is most clearly illustrated by the curcumin analyses. Jazayeri-Tehrani et al. (2019) [62] used nanocurcumin at 80 mg/day, a formulation with considerably enhanced bioavailability, and reported a meaningful reduction in both CRP and HOMA-IR; comparable results were observed for Pourhabibi-Zarandi et al. (2022) and Hellmann et al. (2022) [55,66] at 500 and 400 mg/day, respectively. These observations suggest that bioavailability-adjusted dosing may be a more relevant determinant of response than absolute dose. For omega-3 fatty acids, trials employing alpha-linolenic acid (ALA) used substantially higher doses (10–11 g/day) than those using eicosapentaenoic acid (EPA) and docosahexaenoic acid (DHA) directly (1.8–4.2 g/day), reflecting the recognized inefficiency of ALA-to-EPA/DHA conversion, yet this dose adjustment did not appear to produce systematically different effect sizes.

This review presents methodological and conceptual strengths that distinguish it from prior work. The simultaneous evaluation of four bioactive compounds against the same primary outcomes within a consistently defined population allows a comparative perspective that goes beyond the scope of single-compound analyses, making it possible to directly compare the magnitude of effect across bioactive compounds under a single analytical framework.

Some limitations arise during the analysis. In the search phase, only PubMed and Scopus were searched, so eligible trials indexed exclusively in other databases, such as Embase, the Cochrane Central Register of Controlled Trials (CENTRAL) or Web of Science, may have been missed. Screening and initial data extraction were initially performed by a single reviewer; extraction errors cannot be fully excluded. A first source of methodological complexity was the unit standardization required prior to pooling. CRP values were reported across trials in a variety of units, and conversion to a common scale (CRP, mg/L) was necessary to interpret the magnitude of the effect in clinical terms; all conversions are detailed in Supplementary File S1 for transparency. HOMA-IR presented analogous challenges: where it was not directly reported, it was derived from fasting plasma glucose and fasting insulin using the standard formula.

Several omega-3 trials employed caloric restriction in both groups. As dietary energy restriction independently reduces both systemic inflammation and insulin resistance, such comparators may have attenuated the apparent effect of omega-3 supplementation or alternatively inflated it in trials using ad libitum usual diet as comparator, which may have contributed to the substantial heterogeneity observed.

The clinical heterogeneity of the enrolled populations across all four compounds also constitutes a limitation. While overweight or obesity was a universal inclusion criterion, the co-occurrence of conditions such as PCOS, metabolic syndrome, NAFLD, hypertension, and dyslipidemia varied substantially across trials. These conditions are associated with differing baseline levels of inflammation and insulin resistance and may modulate the responsiveness of these biomarkers to supplementation in ways not captured in the pooled analysis. A subgroup analysis stratified by metabolic phenotype was not feasible given the small number of trials available per compound and outcome. The intervention across trials also varied significantly for all compounds, both in duration and dose, making it difficult to build assumptions regarding effective intervention periods or optimal dosages. Future trials could benefit from standardized formulations, and this should be a priority for future evaluation of the most promising compounds under the scope of this systematic review.

Small-study effects were assessed through visual inspection of residual funnel plots only, as formal statistical tests such as Egger’s test were not performed given the small number of trials included in most of the analyses; while visual inspection is a legitimate method, it is subject to subjectivity and it can reduce sensitivity. Nonetheless, this does not impact the primary analysis. A further limitation concerns the review process itself: title/abstract screening and full-text assessment were performed by a single reviewer, without duplicate independent screening. Lastly, the risk-of-bias rule used for the certainty ratings was defined by the authors, and evaluated under a scope of relative subjectiveness, especially for borderline cases.

The findings highlight several priorities for future investigation. The field would benefit from larger randomized controlled trials with pre-specified sample size calculations based on clinically meaningful effect sizes, particularly for the most promising compounds identified here, curcumin and green tea. A subgroup analysis stratified by metabolic phenotype would allow more precise contextualization of the observed effects, comparing, for example, the response of individuals with NAFLD versus metabolic syndrome versus uncomplicated obesity. Finally, a meta-analysis evaluating dietary patterns naturally rich in these compounds would permit assessment of long-term exposure effects and a meaningful comparison with short-term supplementation.

## 5. Conclusions

This systematic review and meta-analysis evaluated four bioactive compounds–curcumin, green tea extract, resveratrol, and omega-3 fatty acids–against HOMA-IR and CRP in overweight and obese adults, synthesizing 59 randomized controlled trials across eight independent pooled analyses. Of these, only curcumin yielded a statistically significant effect, probably reducing HOMA-IR slightly and representing the most consistent positive finding of the review. Green tea extract produced a borderline reduction in CRP that narrowly missed conventional significance and was accompanied by low certainty of evidence, suggesting a possible but unconfirmed anti-inflammatory trend. Resveratrol and omega-3 supplementation showed no significant effect on either outcome; notably, resveratrol displayed consistently null results with moderate certainty in HOMA IR, pointing to a probable absence of effect rather than merely inconclusive evidence.

In conclusion, curcumin and green tea therefore emerge as the most promising candidates for future investigation. To produce high-quality meta-analyses of genuine scientific relevance, it is essential that well-designed clinical trials be conducted: trials with sufficiently large samples, standardized supplementations of preferably high bioavailability, and standardized statistical analyses appropriate to the research objectives. In this way, future meta-analyses may yield more substantial and reliable findings.

## Figures and Tables

**Figure 1:**
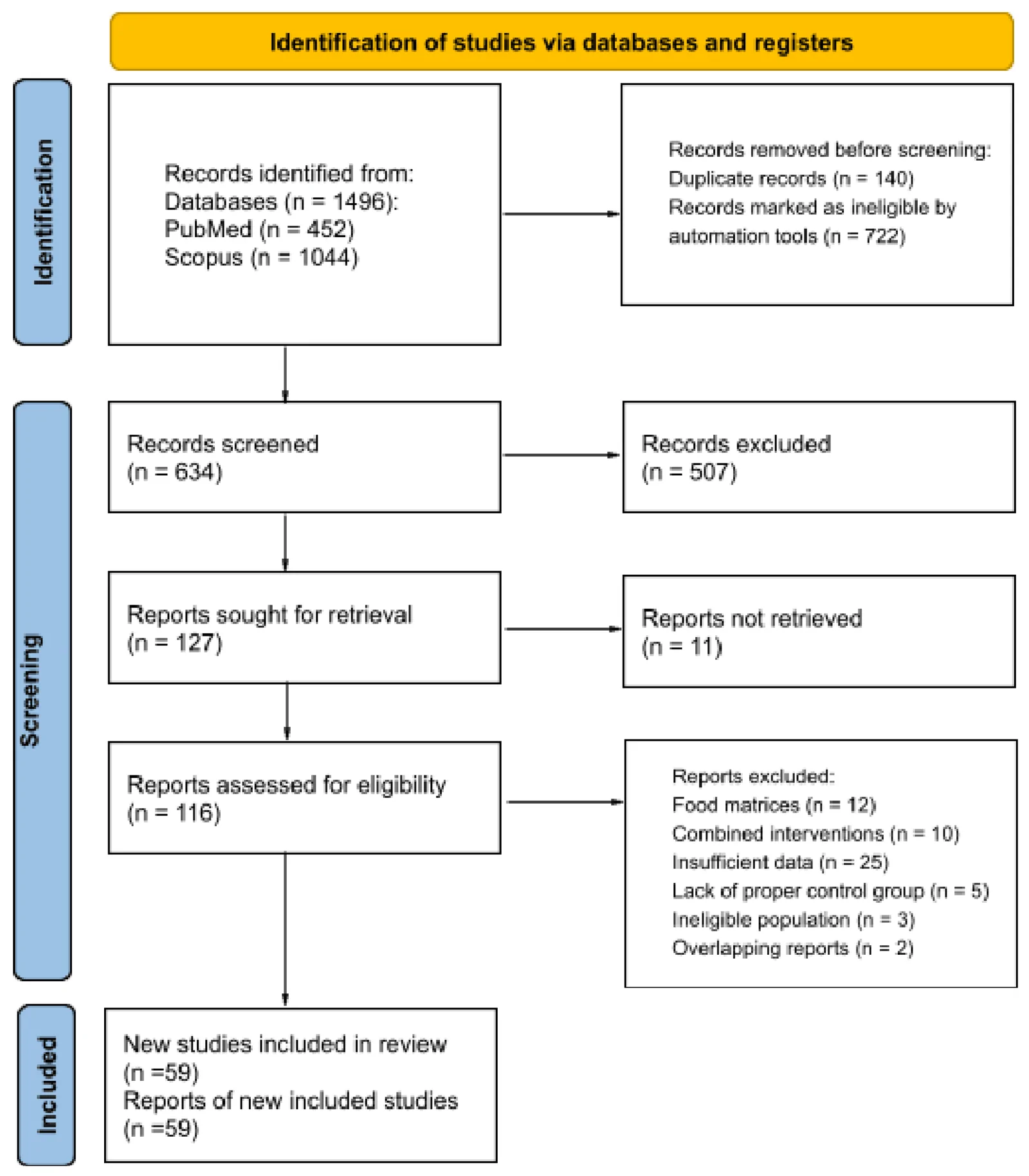
PRISMA (Preferred Reporting Items for Systematic Reviews and Meta-Analyses) flow diagram of study identification, screening, and inclusion. A total of 59 reports were included, categorized by bioactive compounds: Omega-3 (*n* = 23 studies), Curcumin (*n* = 13), Green Tea (*n* = 13 studies), and Resveratrol (*n* = 10).

**Table 1:** Population, Intervention, Comparator, Outcomes, Study design (PICOS) framework of the systematic review. BMI ≥ 25.0 kg/m^2^ is defined as criteria of overweight in adult population. Abbreviations are defined as follows: BMI, body mass index; PUFA, polyunsaturated fatty acid; HOMA-IR, Homeostatic Model Assessment for Insulin Resistance; OGTT, Oral glucose tolerance test; HbA1c, glycated hemoglobin; CRP, C-reactive protein; IL-6, Interleukin 6; and TNF-α, tumor necrosis factor-alpha; RCT, randomized controlled trial.

Component	Description	Operational Definition in This Review
P (Population)	Adults with overweight/obesity	Adults (≥18 years) with BMI ≥ 25.0 kg/m^2^, without diagnosed type 2 diabetes; with or without insulin resistance or metabolic syndrome
I (Intervention)	Bioactive compound supplementation	Oral supplementation with isolated omega-3 PUFA, curcumin, resveratrol, or green tea extract; minimum duration ≥ 4 weeks; no combined structured exercise intervention
C (Comparator)	Placebo or control	Placebo, control supplements, or usual diet; physical activity and caloric restriction interventions must not differ between groups
O (Outcomes)	Glycemic and Inflammatory biomarkers	Primary Outcomes: HOMA-IR Secondary Outcomes: fasting insulin, glucose, OGTT, HbA1c Primary Outcomes: CRP Secondary Outcomes: IL-6, TNF-α, adiponectin
S (Study Design)	Randomized controlled trials	Human RCTs (parallel or crossover design) published in peer-reviewed journals

**Table 2:** Systematic search strategy and strings used in electronic databases. The asterisk (*) denotes truncation, retrieving all terms beginning with the preceding root.

Database	Strings Used	Filters Applied
Pubmed	(“Obesity”[Mesh] OR obes *[tiab] OR “body mass index”[tiab] OR BMI[tiab] OR adiposity[tiab]) AND (“Fatty Acids, Omega-3”[Mesh] OR “omega-3”[tiab] OR “omega 3”[tiab] OR *n*-3[tiab] OR PUFA[tiab] OR EPA[tiab] OR DHA[tiab] OR “Curcumin”[Mesh] OR curcumin[tiab] OR turmeric[tiab] OR “Resveratrol”[Mesh] OR resveratrol[tiab] OR “Tea, Green”[Mesh] OR “green tea”[tiab] OR EGCG[tiab] OR catechin *[tiab]) AND (“Insulin Resistance”[Mesh] OR “insulin resistance”[tiab] OR “insulin sensitivity”[tiab] OR HOMA[tiab] OR HOMA-IR[tiab] OR “fasting glucose”[tiab] OR “fasting insulin”[tiab] OR OGTT[tiab] OR HbA1c[tiab] OR “C-Reactive Protein”[Mesh] OR CRP[tiab] OR “C-reactive protein”[tiab] OR “Interleukin-6”[Mesh] OR IL-6[tiab] OR “interleukin 6”[tiab] OR “Tumor Necrosis Factor-alpha”[Mesh] OR TNF[tiab] OR TNF-α[tiab] OR “tumor necrosis factor”[tiab] OR adiponectin[tiab]) AND (randomized controlled trial[pt] OR controlled clinical trial[pt] OR randomized[tiab] OR randomised[tiab] OR placebo[tiab]) NOT (review[pt] OR meta-analysis[pt] OR “systematic review”[tiab] OR “type 2 diabetes”[tiab] OR “diabetes mellitus type 2”[tiab])	“Article”, “English”
Scopus	TITLE-ABS-KEY (obesity OR obese OR “body mass index” OR BMI) AND TITLE-ABS-KEY (“omega-3” OR “omega 3” OR *n*-3 OR PUFA OR EPA OR DHA OR curcumin OR turmeric OR resveratrol OR “green tea” OR EGCG OR catechin *) AND TITLE-ABS-KEY (“insulin resistance” OR “insulin sensitivity” OR “HOMA-IR” OR “fasting glucose” OR “fasting insulin” OR OGTT OR HbA1c OR “C-reactive protein” OR CRP OR “interleukin 6” OR IL-6 OR “tumor necrosis factor” OR TNF-alpha OR adiponectin) AND TITLE-ABS-KEY (“randomized controlled trial” OR “randomised controlled trial” OR placebo) AND NOT TITLE-ABS-KEY (“type 2 diabetes” OR “diabetes mellitus type 2”)	“Medicine”, “Article”, “English”

**Table 3:** Summary of findings: Resveratrol compared to placebo. Patient or population: adults (≥18 y) with overweight or obesity. Setting: outpatient/community. Intervention: supplement (≥4 weeks). Comparison: placebo. GRADE certainty ratings: ⊕⊕⊕◯ moderate; ⊕⊕◯◯ low. Each ⊕ indicates one level of certainty retained and each ◯ one level of downgrading from high certainty.

Outcome (Follow-Up)	Mean in Placebo Group	Effect with Supplement vs. Placebo (MD, 95% CI)	№ of Participants (Studies)	Certainty of the Evidence (GRADE)	Comments
HOMA-IR (4 wk–6 mo)	3.56 units	MD 0.14 lower (0.35 lower to 0.08 higher)	364 (9 RCTs)	⊕⊕⊕◯ Moderate ^a^	Resveratrol probably results in little to no difference in HOMA-IR.
CRP, mg/L (4–16 wk)	3.34 mg/L	MD 0.37 lower (1.05 lower to 0.30 higher)	308 (7 RCTs)	⊕⊕◯◯ Low ^a,b^	Resveratrol may result in little to no difference in CRP.

^a^ Downgraded one level for risk of bias: most of the weight comes from trials at some concerns or high risk of bias in RoB 2. ^b^ Downgraded one level for imprecision: the 95% CI crosses zero and includes an important effect; fewer than ≈500 participants. Note: № of participants: distinct people (crossover participants counted once). Mean in placebo group: mean end-of-study value in the placebo/control groups of the included trials, weighted by control-group size (a placebo group shared by two arms and crossover participants counted once). CI: confidence interval; CRP: C-reactive protein; HOMA-IR: homeostatic model assessment of insulin resistance; MD: mean difference; RCT: randomized controlled trial.

**Table 4:** Summary of findings: Curcumin compared to placebo. Patient or population: adults (≥18 y) with overweight or obesity. Setting: outpatient/community. Intervention: supplement (≥4 weeks). Comparison: placebo. GRADE certainty ratings: ⊕⊕⊕◯ moderate; ⊕◯◯◯ very low. Each ⊕ indicates one level of certainty retained and each ◯ one level of downgrading from high certainty.

Outcome (Follow-Up)	Mean in Placebo Group	Effect with Supplement vs. Placebo (MD, 95% CI)	№ of Participants (Studies)	Certainty of the Evidence (GRADE)	Comments
HOMA-IR (6–12 wk)	2.98 units	MD 0.30 lower (0.53 to 0.07 lower)	366 (7 RCTs)	⊕⊕⊕◯ Moderate ^a^	Curcumin probably reduces HOMA-IR slightly.
CRP, mg/L (4–12 wk)	11.66 mg/L	MD 0.65 lower (2.44 lower to 1.14 higher)	486 (8 RCTs)	⊕◯◯◯ Very low ^b,c,d^	The evidence is very uncertain about the effect of curcumin on CRP.

^a^ Downgraded one level for imprecision: the 95% CI (−0.53 to −0.07) crosses the threshold of 0.2 SD of the control group (±0.20 HOMA-IR units). ^b^ Downgraded one level for risk of bias: only 28.7% of the weight comes from trials at low risk of bias; 24.3% is at high risk. ^c^ Downgraded one level for inconsistency: *I*^2^ ≈ 79%; precise trials disagree and Amin 2015 is an outlier (without it: MD −0.44, 95% CI −1.44 to 0.55; certainty remains very low). ^d^ Downgraded one level for imprecision: the 95% CI (−2.44 to 1.14) crosses the threshold of 0.2 SD of the control group. Note: № of participants: distinct people (crossover participants counted once). Mean in placebo group: mean end-of-study value in the placebo/control groups of the included trials, weighted by control-group size (a placebo group shared by several arms and crossover participants counted once). The CRP mean is driven by Amin 2015 (28 mg/L). CI: confidence interval; CRP: C-reactive protein; HOMA-IR: homeostatic model assessment of insulin resistance; MD: mean difference; RCT: randomized controlled trial.

**Table 5:** Summary of findings: Green tea extract compared to placebo. Patient or population: adults (≥18 y) with overweight or obesity. Setting: outpatient/community. Intervention: supplement (≥4 weeks). Comparison: placebo. GRADE certainty ratings: ⊕⊕◯◯ low; ⊕◯◯◯ very low. Each ⊕ indicates one level of certainty retained and each ◯ one level of downgrading from high certainty.

Outcome (Follow-Up)	Mean in Placebo Group	Effect with Supplement vs. Placebo (MD, 95% CI)	№ of Participants (Studies)	Certainty of the Evidence (GRADE)	Comments
HOMA-IR (4 wk–12 mo)	3.05 units	MD 0.15 lower (0.63 lower to 0.33 higher)	798 (10 RCTs)	⊕◯◯◯ Very low ^a,b,c^	The evidence is very uncertain about the effect of green tea extract on HOMA-IR.
CRP, mg/L (4 wk–12 mo)	2.93 mg/L	MD 0.43 lower (0.87 lower to 0.02 higher)	409 (7 RCTs)	⊕⊕◯◯ Low ^d,e^	Green tea extract may reduce CRP slightly, but the confidence interval includes no effect.

^a^ Downgraded one level for risk of bias (borderline): 46.6% of the weight comes from trials at low risk of bias. ^b^ Downgraded one level for inconsistency: *I*^2^ ≈ 71%; without Hussain 2017 and Bogdanski 2012 (identified as outliers) sensitivity analysis results in MD 0.10 (95% CI −0.07 to 0.27), τ = 0, and certainty would be high. ^c^ Downgraded one level for imprecision: the 95% CI includes important effects in both directions (±0.30 units). ^d^ Downgraded one level for inconsistency: *I*^2^ ≈ 85%. ^e^ Downgraded one level for imprecision: the 95% CI (*p* = 0.057) includes an important reduction (≤ −0.6 mg/L); fewer than ≈ 800 participants. Note: № of participants: distinct people (crossover participants counted once). Mean in placebo group: mean end-of-study value in the placebo/control groups of the included trials, weighted by control-group size (a placebo group shared by several arms and crossover participants counted once). CI: confidence interval; CRP: C-reactive protein; HOMA-IR: homeostatic model assessment of insulin resistance; MD: mean difference; RCT: randomized controlled trial.

**Table 6:** Summary of findings: Omega-3 fatty acids compared to placebo. Patient or population: adults (≥18 y) with overweight or obesity. Setting: outpatient/community. Intervention: supplement (≥4 weeks). Comparison: placebo. GRADE certainty ratings: ⊕⊕◯◯ low. Each ⊕ indicates one level of certainty retained and each ◯ one level of downgrading from high certainty.

Outcome (Follow-Up)	Mean in Placebo Group	Effect with Supplement vs. Placebo (MD, 95% CI)	№ of Participants (Studies)	Certainty of the Evidence (GRADE)	Comments
HOMA-IR (6 wk–6 mo)	2.76 units	MD 0.04 lower (0.21 lower to 0.14 higher)	1008 (19 RCTs)	⊕⊕◯◯ Low ^a,b^	Omega-3 may result in little to no difference in HOMA-IR.
CRP, mg/L (6 wk–6 mo)	3.36 mg/L	MD 0.16 lower (0.42 lower to 0.11 higher)	767 (15 RCTs)	⊕⊕◯◯ Low ^a,b^	Omega-3 may result in little to no difference in CRP.

^a^ Downgraded one level for risk of bias: little or no weight from trials at low risk of bias (HOMA-IR 16.1%; CRP none). ^b^ Downgraded one level for inconsistency: *I*^2^ ≈ 58% (HOMA-IR) and ≈ 72% (CRP); the most precise trials point in different directions. Note: № of participants: distinct people (crossover participants counted once). Mean in placebo group: mean end-of-study value in the placebo/control groups of the included trials, weighted by control-group size (a placebo group shared by several arms and crossover participants counted once). CI: confidence interval; CRP: C-reactive protein; HOMA-IR: homeostatic model assessment of insulin resistance; MD: mean difference; RCT: randomized controlled trial.

## Data Availability

No new data were created or analyzed in this study. Data sharing is not applicable to this article. The data analyzed in this systematic review and meta-analysis were extracted from previously published studies cited in the reference list. Details of the included studies are provided in Table 3, Table 4, Table 5 and Table 6 and Appendix A.1, Appendix A.2, Appendix A.3 and Appendix A.4. The extracted dataset used for the meta-analysis is available in the Supplementary Materials.

## Supplementary Materials

The following supporting information can be downloaded at: https://www.mdpi.com/article/10.3390/nu18213638/s1, Table S1: Detailed eligibility criteria of the systematic review; File S1: Extracted data and unit conversions applied to each included trial; File S2: RoB 2 assessment of parallel trials; File S3: RoB 2 assessment of crossover trials; File S4: GRADE full evidence profiles; File S5: Full summary of the included studies.

## Abbreviations

The following abbreviations are used in this manuscript:

HOMA-IR	Homeostatic model assessment of insulin resistance
CRP	C-reactive protein
RCT	Randomized controlled trial
BMI	Body mass index
RoB 2	Revised Cochrane risk-of-bias tool for randomized trials
GRADE	Grading of Recommendations Assessment, Development and Evaluation
MD	Mean difference
PROSPERO	International Prospective Register of Systematic Reviews
TNF-α	Tumor necrosis factor-alpha
IL-6	Interleukin-6
IL-1β	Interleukin-1 beta
JNK	c-Jun N-terminal kinase
NF-κB	Nuclear factor kappa B
SIRT1	Sirtuin 1
AMPK	AMP-activated protein kinase
EGCG	Epigallocatechin gallate
IRS-1/IRS-2	Insulin receptor substrate 1 and 2
GLUT-4	Glucose transporter type 4
PRISMA	Preferred Reporting Items for Systematic Reviews and Meta-Analyses
HbA1c	Glycated hemoglobin
PICOS	Population, Intervention, Comparator, Outcomes, Study design
PUFA	Polyunsaturated fatty acid
OGTT	Oral glucose tolerance test
NCBI	National Center for Biotechnology Information
MeSH	Medical Subject Headings
EPA	Eicosapentaenoic acid
DHA	Docosahexaenoic acid
CI	Confidence interval
SD	Standard deviation
REML	Restricted maximum likelihood
PCOS	Polycystic ovary syndrome
NAFLD	Non-alcoholic fatty liver disease
PI	Prediction interval
HOMA2-IR	Updated homeostatic model assessment of insulin resistance
ALA	Alpha-linolenic acid
CENTRAL	Cochrane Central Register of Controlled Trials

## Appendix A

### Appendix A.1

**Table A1:** Summary of Included Studies: Resveratrol. Int = intervention; Ctrl = control; NR = not reported; MetS = metabolic syndrome; IR = Insulin Resistance; NAFLD = non-alcoholic fatty liver disease; Timmers et al., 2011 [47] applied a crossover design; all other studies applied a parallel design.

Study ID	Country	Duration	N Int/Ctrl	Age Int/Ctrl (Years)	BMI Int/Ctrl (kg/m^2^)	Metabolic Status	Daily Dose	Formulation	Primary Outcome
Timmers et al., 2011 [47]	Netherlands	30 d	11/11	52.5 ± 2.1	31.45 ± 0.82 31.59 ± 0.74	Obesity only	150 mg	Capsule	HOMA-IR, CRP
Kantartzis et al., 2018 [67]	Germany	12 wk	53/52	20–60	≥27 ≥27	IR, Obesity	150 mg	Capsule	HOMA-IR, CRP
Arzola-Paniagua et al., 2016 [63]	Mexico	6 mo	15/24	33.7 ± 11.9/ 38.8 ± 9.59	35.6 ± 2.71 34.7 ± 2.89	Obesity only	100 mg	Capsule	HOMA-IR
Poulsen et al., 2013 [68]	Denmark	4 wk	12/12	44.7/ 31.9	32.5 ± 0.6 35.9 ± 1.2	Obesity only	1500 mg	Tablets	HOMA-IR, CRP
Kjær et al., 2017 (150 mg) [42]	Denmark	16 wk	21/24	49.5 ± 0.796	33.4 ± 0.858 34.1 ± 0.77	MetS, Obesity	150 mg	Tablets	HOMA-IR, CRP
Kjær et al., 2017 (1 g) [42]	Denmark	16 wk	21/24	49.5 ± 0.796	33.8 ± 0.668 34.1 ± 0.77	MetS, Obesity	1000 mg	Tablets	HOMA-IR, CRP
de Ligt et al., 2020 [69]	Netherlands	6 mo	20/21	61 ± 1.3/ 62 ± 1.5	30 ± 0.5 29 ± 0.5	Obesity only	150 mg	Capsule	HOMA-IR
Heebøll et al., 2016 [70]	Denmark	6 mo	13/13	43.2/ 43.5	32.1 ± 3.1 32.0 ± 5.4	NAFLD, Overweight/Obese	1500 mg	Tablets	HOMA-IR, CRP
Chachay et al., 2014 [71]	Australia	8 wk	10/10	48.8 ± 12.2/ 47.5 ± 11.2	31.8 31.2	NAFLD, Obesity	3000 mg	Capsule	HOMA-IR, CRP
Faghihzadeh et al., 2014 [72]	Iran	12 wk	25/25	44.04 ± 10.10/46.28 ± 9.52	28.35 ± 3.49 28.75 ± 3.50	NAFLD, Overweight	500 mg	Capsule	CRP
Guevara-Cruz et al., 2026 [99]	Mexico	2 mo	14/18	20–60	34.3 (31.5–36.4) 33.5 (31.2–38.6)	IR, Obesity	150 mg	Capsule	HOMA-IR, CRP

### Appendix A.2

**Table A2:** Summary of Included Studies: Curcumin. Int = intervention; Ctrl = control; NR = not reported; MetS = metabolic syndrome; IR = Insulin Resistance; NAFLD = non-alcoholic fatty liver disease; PCOS = polycystic ovary syndrome. All studies used placebo capsules as control. Mohammadi et al., 2013 [49] and Nieman et al., 2012 [54] applied a crossover design; all other studies applied a parallel design.

Study ID	Country	Length	N Int/Ctrl	Age Int/Ctrl (Years)	BMI Int/Ctrl (kg/m^2^)	Metabolic Status	Daily Dose	Formulation	Primary Outcome
Amin et al., 2015 [48]	Pakistan	8 wk	63/63	42.4 ± 13.7/ 41.57 ± 12.8	28.1 ± 5.0 27.5 ± 4.12	MetS, Overweight	2400 mg	Powdered	CRP
Uchio et al., 2021 [73]	Japan	12 wk	39/40	56.7/ 56.6	26.6 26.4	Overweight	Not clear	Capsules	CRP
Jamilian et al., 2020 [74]	Iran	12 wk	24/26	28.6 ± 4.7/ 27.2 ± 3.4	27.4 ± 3.9 26.4 ± 3.8	PCOS, Overweight	500 mg	Not clear	HOMA-IR
Pourhabibi-Zarandi et al., 2022 [66]	Iran	8 wk	22/22	50.68 ± 9.93/ 50.36 ± 9.70	29.58 ± 5.06 28.94 ± 4.65	Overweight	500 mg	Capsules	HOMA-IR, CRP
Cicero et al., 2020 [53]	Italy	8 wk	40/40	54 ± 3/ 53 ± 5	27.1 ± 1.8 26.9 ± 1.9	IR, Overweight	1600 mg	Tablets	HOMA-IR
Mohammadi et al., 2013 [49]	Iran	30 d	30/30	38.43 ± 10.84	32.60 ± 3.58 32.60 ± 3.58	Obesity	1000 mg	Capsules	CRP
Ghaffari et al., 2018 [100]	Iran	NR	21/21	42.57 ± 6.93/ 40.38 ± 9.26	31.81 ± 4.58 32.92 ± 4.81	NAFLD, Obesity	3000 mg	Capsules	CRP
Campbell et al., 2019 [41]	USA	12 wk	10/10	18–35	>30.0 >30.0	Obesity	500 mg	Pills	HOMA-IR
Nieman et al., 2012 [54]	USA	4 wk	30/30	55.7 ± 1.4	34.5 ± 0.8 34.5 ± 0.8	Obesity	2.8 g	Capsules	CRP
Jazayeri-Tehrani et al., 2019 [62]	Iran	3 mo	42/42	41.8 (5.6) (Int)/42.5 (6.2) (Ctrl)	30.67 (2.14) 30.75 (2.35)	NAFLD, Overweight	80 mg	Capsules	CRP, HOMA-IR
Hellmann et al., 2022 [55]	Denmark	6 wk	18/19	44.8 ± 15.8 (Int)/47.7 ± 12.1 (Ctrl)	38.0 ± 5.5 39.5 ± 6.8	Steatosis, Obesity	400 mg	Tablets	HOMA-IR
Dolati et al., 2020 [75]	Iran	8 wk	10/10	38.9 ± 5.40 (Int)/40.80 ± 3.55 (Ctrl)	26.96 ± 1.90 27.21 ± 1.43	Overweight	500 mg	Capsules	HOMA-IR
Sohaei et al., 2019 [101]	Iran	6 wk	27/24	29.58 ± 5 (Int)/29.40 ± 5.33 (Ctrl)	29.67 ± 3.72 31.32 ± 4.6	PCOS, Obesity	500 mg	Pellets	HOMA-IR, CRP

### Appendix A.3

**Table A3:** Summary of Included Studies: Green Tea. Int = intervention; Ctrl = control MetS = metabolic syndrome; NAFLD = non-alcoholic fatty liver disease; PCOS = polycystic ovary syndrome; All studies used placebo capsules as control. Zeng et al., 2024 [65] and Nogueira et al., 2017 [64] applied a crossover design; all other studies applied a parallel design.

Study ID	Country	Length	N Int/Ctrl	Age (Years)	BMI Int/Ctrl (kg/m^2^)	Metabolic Status	Daily Dose	Formulation	Primary Outcome
Rondanelli et al., 2022 [52]	Italy	60 d	14/14	60.57 ± 7.28/ 56.92 ± 5.70	31.99 ± 2.23 31.10 ± 3.54	Obesity	150 mg	Tablets	HOMA-IR, CRP
Zeng et al., 2024 [65]	USA	4 wk	21/21	40 ± 3	35.2 ± 1.0	MetS, Obesity	890 mg	Confections	HOMA-IR
Hsu et al., 2008 [76]	Taiwan	12 wk	41/37	43.0 ± 11.1/ 43.9 ± 12.6	31.2 (3.5) 30.5 (4.6)	Obesity	400 mg	Capsules	HOMA-IR
Mielgo-Ayuso et al., 2014 [56]	Spain	12 wk	43/40	19–49 years	33.7 ± 2.6 34.3 ± 3.0	Obesity	300 mg	Capsules	HOMA-IR, CRP
Brown et al., 2009 [77]	UK	8 wk	46/42	52.15 ± 6.43/ 50.57 ± 6.48	31.21 ± 2.80 30.96 ± 2.45	Obesity	800 mg	Capsules	HOMA-IR
Cunningham et al., 2026 [57]	USA	12 mo	50/47	60.4 ± 5.2/ 60.4 ± 5.5	29.1 ± 3.1 28.7 ± 2.7	Overweight	843 mg	Capsules	CRP
Dostal et al., 2016 [93]	USA	12 mo	117/120	60.9 ± 0.45/ 60.6 ± 0.47	28.5 ± 0.28 27.9 ± 0.25	Overweight and Obesity	1315 mg	Capsules	HOMA-IR
Bogdanski et al., 2012 [51]	Poland	3 mo	28/28	49.2 ± 8.8/ 51.5 ± 7.4	32.5 ± 3.3 33.9 ± 2.3	Hypertension, Obesity	379 mg	Capsules	HOMA-IR, CRP
Nogueira et al., 2017 [64]	Brazil	4 wk	20/20	41.1 (8.4)	33.56 ± 0.98	Prehypertension, Obesity	1.5 g	Capsules	CRP
Taghizadeh et al., 2017 [59]	Iran	8 wk	25/25	32.2 ± 6.9/ 35.1 ± 7.2	32.3 ± 4.2 32.4 ± 5.9	Obesity	500 mg	Capsules	HOMA-IR
Mombaini et al., 2017 [58]	Iran	45 d	22/23	23.22 ± 5.24/ 24.17 ± 6.83	28.96 ± 6.93 28.90 ± 4.30	PCOS, Overweight	500 mg	Tablets	CRP
Hussain et al., 2017 [50]	Pakistan	12 wk	40/40	25 ± 18/ 28 ± 15	29.5 ± 2.2 28.6 ± 0.6	NAFLD, Overweight	1000 mg	Capsules	HOMA-IR, CRP
Chen et al., 2016 [78]	Taiwan	12 wk	39/38	44.1 ± 10.9/ 44.9 ± 11.9	31.0 ± 3.8 30.0 ± 3.5	Obesity	856.8 mg	Capsules	HOMA-IR

### Appendix A.4

**Table A4:** Summary of Included Studies: Omega-3. Albert et al., 2015 and Jellema et al., 2004 applied a crossover design; all other studies applied a parallel design. Int = intervention; Ctrl = control; NR = not reported; MetS = metabolic syndrome; IR = Insulin Resistance; NAFLD = non-alcoholic fatty liver disease.

Study ID	Country	Length	N Int/Ctrl	Age Int/Ctrl (Years)	BMI Int/Ctrl (kg/m^2^)	Metabolic Status	Daily Dose	Formulation	Control	Primary Outcome
H-Y Su et al., 2015 [94]	Taiwan	12 wk	35/36	NR	29.3 ± 3.1 29.9 ± 4.6	MetS, Obesity	1280 mg EPA + 850 mg DHA	Capsules	Control diet	HOMA-IR
Krebs et al., 2006 [79]	UK	24 wk	35/32	44.7 ± 13.2	35.0 ± 5.5 35.0 ± 5.5	IR, Obesity	1300 mg EPA + 2900 mg DHA	Capsule	Placebo	HOMA-IR, CRP
Nelson et al., 2007 [80]	USA	8 wk	30/27	38.80 ± 11.97/38.15 ± 10.77	30.19 ± 5.50 30.46 ± 4.56	Obesity	11 g ALA	Capsule	Usual diet	HOMA-IR
Simão et al., 2012 [81]	Brazil	90 d	19/15	47.9 ± 9.98	32.93 35.36	MetS, Obesity	3 g *n*-3 (non specified)	Capsule	Usual diet	CRP, HOMA-IR
Schwab et al., 2018 [82]	Finland	12 wk	18/20	58.9 ± 6.5	29.2 ± 2.4 29.2 ± 2.4	IR, Overweight/Obesity	10 g ALA	Oil	Control diet	HOMA-IR, CRP
Munro & Garg, 2012 [95]	Australia	14 wk	17/12	40.5 ± 10.9/42.3± 9.1	32.7 ± 3.28 33.0 ± 3.17	Obesity	420 mg EPA + 1620 mg DHA	Capsule	Placebo	CRP
Chan et al., 2002 [83]	Australia	6 wk	12/12	53.5 ± 9.0	33.6 ± 4.1	Obesity	4 g *n*-3 (non specified)	Capsule	Placebo	HOMA-IR, CRP
Razny et al., 2015 [84]	Poland	3 mo	24/24	47 ± 2	34.25 ± 0.7 35.24 ± 0.72	Obesity	1.8 g *n*-3 (DHA/EPA, 5:1)	Capsules	Placebo	HOMA-IR
de Luis et al., 2016 [96]	Spain	6 mo	14/15	47.4 ± 9.1/44.3 ± 11.7	33.4 ± 1.4 32.95 ± 1.9	Obesity	500 mg *n*-3 (250 mg DHA)	Capsules	Placebo	HOMA-IR, CRP
Rajkumar et al., 2014 [85]	India	6 wk	15/15	49	28.79	Overweight	180 mg EPA + 120 mg DHA	Capsules	Placebo	HOMA-IR, CRP
Simão et al., 2014 [86]	Brazil	90 d	19/15	47.9 ± 9.98	34.3 ± 7.2 36.3 ± 6.5	MetS, Obesity	1800 mg EPA + 1200 mg DHA	Capsules	Usual diet	HOMA-IR
Yang et al., 2019 (DHA + EPA) [44]	China	12 wk	35/34	55.09 ± 7.47/58.38 ± 6.87	26.92 ± 2.72 26.78 ± 2.98	Hypertension, Overweight	2 g *n*-3 (DHA + EPA)	Capsules	Control oil	HOMA-IR
Yang et al., 2019 (ALA) [44]	China	12 wk	39/34	56.68 ± 7.83/58.38 ± 6.87	26.83 ± 3.06 26.78 ± 2.98	Hypertension, Overweight	2.5 g ALA	Capsules	Control oil	HOMA-IR
Derosa et al., 2012 [87]	Italy	6 mo	78/79	≥18 and ≤75	26.0 ± 1.3 27.2 ± 1.9	Dyslipidemia, Overweight	1200 mg EPA + 1350 mg DHA	Capsules	Placebo	HOMA-IR, CRP
Tardivo et al., 2015 [88]	Brazil	6 mo	44/43	55.1 ± 6.6/55.0 ± 7.3	32.8 ± 4.7 32.0 ± 4.6	MetS, Obesity	900 mg *n*-3 (non specified)	Capsules	Control diet	HOMA-IR, CRP
Qin et al., 2015 [89]	China	3 mo	36/34	46.0 ± 10.68/44.3 ± 10.90	26.4 ± 3.9 26.0 ± 2.8	NAFLD, hyperlipidemia, Overweight	4 g “fish oil”	Capsules	Placebo	HOMA-IR
Shabrina et al., 2020 [90]	Taiwan	12 wk	9/12	50.4 ± 11.2/43.4 ± 14.6	30.3 ± 3.74 31.8 ± 5.09	MetS, Obesity	1280 mg EPA + 850 mg DHA	Capsules	Control diet	HOMA-IR, CRP
Ebrahimi et al., 2009 [97]	Iran	6 mo	47/42	53.5 ± 12.7/52.3 ± 11.1	30.3 ± 5.2 30.4 ± 6.1	MetS, Obesity	1 g fish oil (180 mg EPA + 120 mg DHA)	Capsules	Control diet	CRP
Mori et al., 2000 (EPA) [43]	Australia	6 wk	19/20	48.8 ± 1.1	29.0 ± 0.7 28.4 ± 0.5	Overweight	4 g EPA	Capsules	Placebo	HOMA-IR
Mori et al., 2000 (DHA) [43]	Australia	6 wk	17/20	48.8 ± 1.1	28.9 ± 0.7 28.4 ± 0.5	Overweight	4 g DHA	Capsules	Placebo	HOMA-IR
Albert et al., 2015 [60]	Australia	8 wk	47/47	46.5 ± 5.1	27.4 ± 1.8	Overweight	230 mg EPA + 154 mg DHA	Capsules	Placebo	HOMA-IR, CRP
Wong et al., 2013 [91]	Australia	16 wk	13/12	60 ± 4	34 ± 2 33 ± 1	Obesity	4 g *n*-3 (non specified)	Capsules	Control diet	HOMA-IR
Huerta et al., 2016 [98]	Spain	10 wk	16/21	20–50	27.5–40 27.5–40	Obesity	1300 mg EPA	Capsules	Placebo	CRP
Razny et al., 2021 [92]	Poland	3 mo	13/14	41.0 ± 9.9	30.84 ± 3.48 31.56 ± 3.75	Obesity	1.8 g *n*-3 (non specified)	Capsules	Placebo	HOMA-IR
Jellema et al., 2004 [61]	Netherlands	6 wk	11/11	59 ± 9	31.3 ± 1.82 31.3 ± 2.08	Obesity	600 mg EPA + 510 mg DHA	Capsules	Placebo	HOMA-IR, CRP
