# Supplementary material for: Effects of Bioactive Compound Supplementation on Insulin Resistance and Systemic Inflammation in Overweight and Obese Adults: A Systematic Review and Meta-Analysis of Randomized Controlled Trials
Source: Nutrients. 2026 Oct 31;18(21):3638. doi: 10.3390/nu18213638 (PMC13765097; doi:10.3390/nu18213638)
Supplement: Supplementary file 1 [file nutrients-18-03638-s001.zip › File S4. GRADE full evidence profiles.docx.pdf]

File S4. GRADE full evidence profiles.

Bioactive compounds versus placebo on HOMA-IR and CRP. Estimates are those of the final analyses accounting for dependency (crossover trials and shared placebo groups). Certainty starts HIGH for randomised trials and was downgraded for risk of bias, inconsistency, indirectness, imprecision and publication bias; no upgrading was applied.

Table S2. GRADE evidence profile: Resveratrol supplementation (≥ 4 weeks) compared to placebo

Population: adults (≥ 18 y) with overweight or obesity (some with associated conditions: NAFLD, MetS, PCOS, IR). Setting: outpatient / community (randomised controlled trials). Comparison: placebo.

				**Certainty assessment**					**№ of participants**		**Effect**			
**Outcome**	**Follow-up**	**№ of studies**	**Study design**	**Risk of bias**	**Inconsistency**	**Indirectness**	**Imprecision**	**Other considerations**	**Intervention**	**Placebo**	**Relative effect (95% CI)**	**Absolute effect (95% CI)**	**Certainty**	**Importance**
**HOMA-IR**	4 wk – 6 mo	9	RCT	Serious[a]	Not serious[b]	Not serious[c]	Not serious[d]	None	190	185	—	MD 0.14 units lower (0.35 lower to 0.08 higher)	⊕⊕⊕◯ **Moderate**	Critical
**CRP (mg/L)**	4 – 16 wk	7	RCT	Serious[e]	Not serious[f]	Not serious[c]	Serious[g]	None	167	152	—	MD 0.37 mg/L lower (1.05 lower to 0.30 higher)	⊕⊕◯◯ **Low**	Critical

Explanations

a. Risk of bias: 98.5% of the weight comes from trials at some concerns (86.0%) or high risk of bias (12.4%) in RoB 2 (mainly randomisation/allocation concealment, missing outcome data and completers-only analyses); only 1.5% is low risk.

b. Inconsistency: not serious; Q(9) = 7.25, p = 0.611; $\tau = 0.15$; $I^2 \approx 0\%$. Confidence intervals overlap widely.

c. Indirectness: not serious; PICO matched. Wide dose (150 mg – 3 g/day) and duration ranges, without a dose–response signal.

d. Imprecision: not serious. The 95% CI (−0.35 to 0.08) crosses zero but stays within the threshold of 0.2 SD of the control group (±0.39 HOMA-IR units)

e. Risk of bias: 86.5% of the weight comes from trials at some concerns (78.0%) or high risk of bias (8.5%); only 13.5% is low risk (Chachay 2014).

f. Inconsistency: not serious; Q(7) = 8.49, p = 0.292; $\tau = 0.46$; $I^2 \approx 17\%$. Point estimates fall on both sides of zero but all confidence intervals overlap.

g. Imprecision: the 95% CI (−1.05 to 0.30 mg/L) crosses zero and includes an important reduction (≤ −0.6 mg/L); ≈ 308 distinct participants, below the optimal information size.

Table S3. GRADE evidence profile: Curcumin supplementation (≥ 4 weeks) compared to placebo

Population: adults (≥ 18 y) with overweight or obesity (some with associated conditions: NAFLD, MetS, PCOS, IR). Setting: outpatient / community (randomised controlled trials). Comparison: placebo.

		Certainty assessment							№ of participants		Effect			
Outcome	Follow-up	№ of studies	Study design	Risk of bias	Inconsistency	Indirectness	Imprecision	Other considerations	Intervention	Placebo	Relative effect (95% CI)	Absolute effect (95% CI)	Certainty	Importance
**HOMA-IR**	6 – 12 wk	7	RCT	Not serious[a]	Not serious[b]	Not serious[c]	Serious[d]	None	183	183	—	MD 0.30 units lower (0.53 lower to 0.07 lower)	⊕⊕⊕◯ **Moderate**	Critical
**CRP (mg/L)**	4 – 12 wk	8	RCT	Serious[e]	Serious[f]	Not serious[c]	Serious[g]	None	274	272	—	MD 0.65 mg/L lower (2.44 lower to 1.14 higher)[h]	⊕◯◯◯ **Very low**	Critical

Explanations

a. Risk of bias: 72.3% of the weight comes from trials at low risk of bias; the rest is at some concerns (27.7%); no trial at high risk.

b. Inconsistency: not serious; $Q(6) = 7.53$, $p = 0.275$; $\tau = 0.13$; $I^2 \approx 20\%$. All point estimates are at or below zero and the confidence intervals overlap.

c. Indirectness: not serious; PICO matched. Formulations (extract, phytosome, lecithin, nano-curcumin) and doses (80–1000 mg/day) vary.

d. Imprecision: the 95% CI excludes zero, but the number of participants (366) is below the optimal information size (≈ 800) and the upper limit is close to no effect; the result leans on a single heavy trial (Jazayeri-Tehrani).

e. Risk of bias: only 28.7% of the weight comes from trials at low risk of bias; 47.0% is at some concerns and 24.3% at high risk (heaviest trial: per-protocol analysis with outcome-based exclusions; two crossover trials without carryover/period information).

f. Inconsistency: $Q(7) = 32.74$, $p < 0.001$; $\tau = 0.91$; $I^2 \approx 79\%$. Precise trials disagree with non-overlapping confidence intervals (Uchio −0.24 vs Jazayeri-Tehrani −1.60), and Amin 2015 (−14.00 [−20.66, −7.34]) does not overlap any other trial; heterogeneity is unexplained.

g. Imprecision: the 95% CI (−2.44 to 1.14 mg/L) crosses zero and includes both an important reduction and an important increase (±0.6 mg/L); ≈ 465 distinct participants, below the optimal information size.

h. Sensitivity analysis excluding the outlying Amin 2015 trial (7 trials): MD −0.44 (95% CI −1.44 to 0.55); certainty remains very low.

dence in the effect estimate: the true effect is likely to be substantially different from the estimate of the effect.

Table S4. GRADE evidence profile: Green tea extract supplementation (≥ 4 weeks) compared to placebo

Population: adults (≥ 18 y) with overweight or obesity (some with associated conditions: NAFLD, MetS, PCOS, IR). Setting: outpatient / community (randomised controlled trials). Comparison: placebo.

Outcome	Follow-up	Certainty assessment							№ of participants		Effect		Certainty	Importance
		№ of studies	Study design	Risk of bias	Inconsistency	Indirectness	Imprecision	Other considerations	Intervention	Placebo	Relative effect (95% CI)	Absolute effect (95% CI)		
**HOMA-IR**	4 wk – 12 mo	10	RCT	Serious[a]	Serious[b]	Not serious[c]	Serious[d]	None	414	405	—	MD 0.15 units lower (0.63 lower to 0.33 higher)[h]	⊕◯◯◯ **Very low**	Critical
**CRP (mg/L)**	4 wk – 12 mo	7	RCT	Not serious[e]	Serious[f]	Not serious[c]	Serious[g]	None	210	207	—	MD 0.43 mg/L lower (0.87 lower to 0.02 higher)	⊕⊕◯◯ **Low**	Critical

Explanations

a. Risk of bias: 46.6% of the weight comes from trials at low risk of bias (just below the 50% threshold), 48.3% at some concerns and 5.1% at high risk; borderline judgement.

b. Inconsistency: $Q(9) = 31.00$, $p < 0.001$; $\tau = 0.53$; $I^2 \approx 71\%$. Hussain (−1.66) and Bogdanski (−0.80) do not overlap Dostal, Mielgo-Ayuso or Brown; heterogeneity is unexplained.

c. Indirectness: not serious; PICO matched (one trial used a combined preparation; excluding it does not change the estimate).

d. Imprecision: the 95% CI (−0.63 to 0.33) crosses zero and includes important effects in both directions (±0.30 units), although the number of participants (≈ 790 distinct) is close to the optimal information size (≈ 800).

e. Risk of bias: 53.2% of the weight comes from trials at low risk of bias (the remainder at some concerns); no trial at high risk.

f. Inconsistency: $Q(6) = 40.41$, $p < 0.001$; $\tau = 0.42$; $I^2 \approx 85\%$. The most precise trials disagree with non-overlapping confidence intervals (Hussain −1.03 vs Rondanelli −0.13); unexplained.

g. Imprecision: the 95% CI (−0.87 to 0.02 mg/L) reaches zero ($p = 0.057$) and includes an important reduction (≤ −0.6 mg/L); ≈ 397 distinct participants, below the optimal information size.

h. Sensitivity analysis for HOMA-IR excluding the two influential trials (Hussain 2017 and Bogdanski 2012; 8 trials, ≈ 654 distinct participants): MD 0.10 (95% CI −0.07 to 0.27); $\tau = 0$, $I^2 = 0\%$. Applying the same rules, risk of bias (57.8% of the weight at low risk), inconsistency and imprecision would not be serious, and certainty would be High.

very little confidence in the effect estimate: the true effect is likely to be substantially different from the estimate of the effect.

Table S5. GRADE evidence profile: Omega-3 fatty acid supplementation (≥ 4 weeks) compared to placebo

Population: adults (≥ 18 y) with overweight or obesity (some with associated conditions: NAFLD, MetS, PCOS, IR). Setting: outpatient / community (randomised controlled trials). Comparison: placebo.

Outcome	Follow-up	Certainty assessment							№ of participants		Effect		Certainty	Importance
		№ of studies	Study design	Risk of bias	Inconsistency	Indirectness	Imprecision	Other considerations	Intervention	Placebo	Relative effect (95% CI)	Absolute effect (95% CI)		
**HOMA-IR**	6 wk – 6 mo	19	RCT	Serious[a]	Serious[b]	Not serious[c]	Not serious[d]	None	563	503	—	MD 0.04 units lower (0.21 lower to 0.14 higher)	⊕⊕○○ **Low**	Critical
**CRP (mg/L)**	6 wk – 6 mo	15	RCT	Serious[e]	Serious[f]	Not serious[c]	Not serious[g]	None	416	409	—	MD 0.16 mg/L lower (0.42 lower to 0.11 higher)	⊕⊕○○ **Low**	Critical

Explanations

a. Risk of bias: only 16.1% of the weight comes from trials at low risk of bias; 72.4% is at some concerns (randomisation, deviations and missing data) and 11.5% at high risk.

b. Inconsistency: $Q(20) = 47.60$, $p < 0.001$; $\tau = 0.29$; $I^2 \approx 58\%$. Precise trials point in opposite directions (Rajkumar −0.42 and Derosa −0.38 versus Mori EPA +0.45, Mori DHA +0.61 and de Luis +0.40); unexplained.

c. Indirectness: not serious; PICO matched (ALA, EPA and DHA formulations; balanced co-interventions in one trial).

d. Imprecision: not serious; ≈ 1,008 distinct participants (above the optimal information size) and a narrow 95% CI (−0.21 to 0.14) that excludes important effects in either direction (±0.30 units).

e. Risk of bias: no trial at low risk of bias; 89.8% of the weight is at some concerns and 10.2% at high risk.

f. Inconsistency: $Q(14) = 50.91$, $p < 0.001$; $\tau = 0.28$; $I^2 \approx 72\%$. The two most precise trials disagree (Derosa −0.60 [−0.72, −0.48] versus Krebs −0.08 [−0.22, 0.06]); unexplained.

g. Imprecision: not serious; ≈ 767 distinct participants (above the optimal information size); the 95% CI (−0.42 to 0.11 mg/L) crosses zero but excludes an important effect in either direction (±0.6 mg/L).

General notes. Pooled effects: random-effects meta-analysis (REML) with Knapp–Hartung adjustment; MD = intervention minus placebo (negative = lower with the supplement). Crossover trials were analysed with a paired-data correction (standard error × $\sqrt{(1 - r)}$, $r = 0.5$; $r = 0.8$ in sensitivity analyses) and the placebo group of multi-arm trials was split among the intervention arms (Cochrane Handbook, chapter 23). Participants: crossover participants are counted in both columns; a placebo group shared by several intervention arms is counted once. Publication bias was not formally tested (fewer than 10 studies in most analyses); funnel plots were inspected and no downgrade was made. Risk of bias was judged from the share of meta-analytic weight by overall RoB 2 category (≥ 50% Low = not serious; ≥ 50% High = very serious; otherwise serious); Important effects for imprecision were defined as 0.2 SD of the control group (small effect), and the optimal information size (≈ 800 participants for this effect) was considered only when the CI did not cross the threshold and the effect was large. Number of studies refers to distinct trials; effect sizes entered may be more numerous where a trial contributed two comparisons.

CI: confidence interval; CRP: C-reactive protein / high-sensitivity CRP (mg/L); HOMA-IR: homeostatic model assessment of insulin resistance (fasting glucose × insulin / 22.5); MD: mean difference; RCT: randomised controlled trial; GRADE: Grading of Recommendations Assessment, Development and Evaluation.

GRADE Working Group grades of evidence

High: We are very confident that the true effect lies close to that of the estimate of the effect.

Moderate: We are moderately confident in the effect estimate: the true effect is likely to be close to the estimate of the effect, but there is a possibility that it is substantially different.

Low: Our confidence in the effect estimate is limited: the true effect may be substantially different from the estimate of the effect.

Very low: We have very little confidence in the effect estimate: the true effect is likely to be substantially different from the estimate of the effect.
