# Supplementary material for: Effects of Bioactive Compound Supplementation on Insulin Resistance and Systemic Inflammation in Overweight and Obese Adults: A Systematic Review and Meta-Analysis of Randomized Controlled Trials
Source: Nutrients. 2026 Oct 31;18(21):3638. doi: 10.3390/nu18213638 (PMC13765097; doi:10.3390/nu18213638)
Supplement: Supplementary file 1 [file nutrients-18-03638-s001.zip › File S5. Full summary of the included studies.pdf]

File S5. Full summary of the included studies.

Table S6. Summary of Included Studies: Resveratrol. Int = Intervention; Ctrl = Control; MetS = Metabolic Syndrome; IR = Insulin Resistance; NAFLD = Non-Alcoholic Fatty Liver Disease. All studies used placebo capsules or tablets as control. Data extracted directly from original publications, as reported.

Study ID	Country	Design	Durati-on	N Int	N Ctrl	Age (years) (Int/Ctrl)	BMI Int (kg/m²)	BMI Ctrl (kg/m²)	Metabolic Status	Daily Dose	Formula-tion	Primary Outcome
Timmers et al., 2011	Netherlands	Crossover	30 d	11	11	52.5 ± 2.1	31.45 ± 0.82	31.59 ± 0.74	Obesity only	150 mg	Capsule	HOMA-IR, CRP
Kantartzis et al., 2018	Germany	Parallel	12 wk	53	52	20–60	≥ 27	≥ 27	IR, Obesity	150 mg	Capsule	HOMA-IR, CRP
Arzola-Paniagua et al., 2016	Mexico	Parallel	6 mo	15	24	33.7 ± 11.9 / 38.8 ± 9.59	35.6 ± 2.71	34.7 ± 2.89	Obesity only	100 mg	Capsule	HOMA-IR
Poulsen et al., 2013	Denmark	Parallel	4 wk	12	12	44.7 / 31.9	32.5 ± 0.6	35.9 ± 1.2	Obesity only	1500 mg	Tablets	HOMA-IR, CRP
Kjær et al., 2017 (150 mg)	Denmark	Parallel	16 wk	21	24	49.5 ± 0.796	33.4 ± 0.858	34.1 ± 0.77	MetS, Obesity	150 mg	Tablets	HOMA-IR, CRP
Kjær et al., 2017 (1 g)	Denmark	Parallel	16 wk	21	24	49.5 ± 0.796	33.8 ± 0.668	34.1 ± 0.77	MetS, Obesity	1000 mg	Tablets	HOMA-IR, CRP
de Ligt et al., 2020	Netherlands	Parallel	6 mo	20	21	61 ± 1.3 / 62 ± 1.5	30 ± 0.5	29 ± 0.5	Obesity only	150 mg	Capsule	HOMA-IR
Heebøll et al., 2016	Denmark	Parallel	6 mo	13	13	43.2 / 43.5	32.1 ± 3.1	32.0 ± 5.4	NAFLD, Overweight/Obese	1500 mg	Tablets	HOMA-IR, CRP
Chachay et al., 2014	Australia	Parallel	8 wk	10	10	48.8 ± 12.2 / 47.5 ± 11.2	31.8	31.2	NAFLD, Obesity	3000 mg	Capsule	HOMA-IR, CRP
Faghihzadeh et al., 2014	Iran	Parallel	12 wk	25	25	44.04 ± 10.10 / 46.28 ± 9.52	28.35 ± 3.49	28.75 ± 3.50	NAFLD, Overweight	500 mg	Capsule	CRP

Study ID	Country	Design	Durati-on	N Int	N Ctrl	Age (years) (Int/Ctrl)	BMI Int (kg/m²)	BMI Ctrl (kg/m²)	Metabolic Status	Daily Dose	Formula-tion	Primary Outcome
Guevara-Cruz et al., 2026	Mexico	Parallel	2 mo	14	18	20–60	34.3 (31.5- 36.4)	33.5 (31.2- 38.6)	IR, Obesity	150 mg	Capsule	HOMA-IR, CRP

Table S7. Summary of Included Studies: Curcumin. Int = Intervention; Ctrl = Control; NR = Not Reported; MetS = Metabolic Syndrome; IR = Insulin Resistance; NAFLD = Non-Alcoholic Fatty Liver Disease; PCOS = Polycystic Ovary Syndrome. All studies used placebo capsules or tablets as control. Data extracted directly from original publications, as reported, and units standardized.

Study ID	Country	Design	Duration	N Int	N Ctrl	Age (years) (Int/Ctrl)	Int BMI (kg/m²)	Ctrl BMI (kg/m²)	Metabolic Status	Daily Dose	Formulation	Primary Outcome
Amin et al., 2015	Pakistan	Parallel	8 wk	63	63	42.4 ± 13.7 / 41.57 ± 12.8	28.1 ± 5.0	27.5 ± 4.12	MetS, Overweight	2400 mg	Powdered	CRP
Uchio et al., 2021	Japan	Parallel	12 wk	39	40	56.7 / 56.6	26.6	26.4	Overweight	Not clear	Capsules	CRP
Jamilian et al., 2020	Iran	Parallel	12 wk	24	26	28.6 ± 4.7 / 27.2 ± 3.4	27.4 ± 3.9	26.4 ± 3.8	PCOS, Overweight	500 mg	Not clear	HOMA-IR
Pourhabibi-Zarandi et al., 2022	Iran	Parallel	8 wk	22	22	50.68 ± 9.93 / 50.36 ± 9.70	29.58 ± 5.06	28.94 ± 4.65	Overweight	500 mg	Capsules	HOMA-IR, CRP
Cicero et al., 2020	Italy	Parallel	8 wk	40	40	54 ± 3 / 53 ± 5	27.1 ± 1.8	26.9 ± 1.9	IR, Overweight	1600 mg	Tablets	HOMA-IR
Mohammadi et al., 2013	Iran	Crossover	30 d	30	30	38.43 ± 10.84	32.60 ± 3.58	32.60 ± 3.58	Obesity	1000 mg	Capsules	CRP
Ghaffari et al., 2018	Iran	Parallel	NR	21	21	42.57 ± 6.93 / 40.38 ± 9.26	31.81 ± 4.58	32.92 ± 4.81	NAFLD, Obesity	3000 mg	Capsules	CRP
Campbell et al., 2019	USA	Parallel	12 wk	10	10	18–35	> 30.0	> 30.0	Obesity	500 mg	Pills	HOMA-IR
Nieman et al., 2012	USA	Crossover	4 wk	30	30	55.7 ± 1.4	34.5 ± 0.8	34.5 ± 0.8	Obesity	2800 g	Capsules	CRP
Jazayeri-Tehrani et al., 2019	Iran	Parallel	3 mo	42	42	41.8 ± 5.6 / 42.5 ± 6.2	30.67 ±2.14	30.75 ±2.35	NAFLD, Overweight	80 mg	Capsules	CRP, HOMA-IR
Hellmann et al., 2022	Denmark	Parallel	6 wk	18	19	44.8 ± 15.8 / 47.7 ± 12.1	38.0 ± 5.5	39.5 ± 6.8	Steatosis, Obesity	400 mg	Tablets	HOMA-IR

Study ID	Country	Design	Duration	N Int	N Ctrl	Age (years) (Int/Ctrl)	Int BMI (kg/m²)	Ctrl BMI (kg/m²)	Metabolic Status	Daily Dose	Formulation	Primary Outcome
Dolati et al., 2020	Iran	Parallel	8 wk	10	10	38.9 ± 5.40/ 40.80 ± 3.55	26.96 ± 1.90	27.21 ± 1.43	Overweight	500 mg	Capsules	HOMA-IR
Sohaei et al., 2019	Iran	Parallel	6 wk	27	24	29.58 ± 5/ 29.40 ± 5.33	29.67 ± 3.72	31.32 ± 4.6	PCOS, Obesity	500 mg	Pellets	HOMA-IR, CRP

Table S8. Summary of Included Studies: Gree Tea. Int = Intervention; Ctrl = Control; MetS = Metabolic Syndrome; NAFLD = Non-Alcoholic Fatty Liver Disease; PCOS = Polycystic Ovary Syndrome. All studies used placebo capsules or tablets as control. Data extracted directly from original publications, as reported, and units standardized.

Study ID	Country	Design	Duration	N Int	N Ctrl	Age (years) (Int/Ctrl)	BMI Int (kg/m²)	BMI Ctrl (kg/m²)	Metabolic Status	Daily Dose	Formulation	Primary Outcome
Rondanelli et al., 2022	Italy	Parallel	60 d	14	14	60.57 ± 7.28) / 56.92 ± 5.70	31.99 ± 2.23	31.10 ± 3.54	Obesity	150 mg	Tablets	HOMA-IR, CRP
Zeng et al., 2024	USA	Crossover	4 wk	21	21	40 ± 3	35.2 ± 1.0	35.2 ± 1.0	MetS, Obesity	890 mg	Confections	HOMA-IR
Hsu et al., 2008	Taiwan	Parallel	12 wk	41	37	43.0 ± 11.1 / 43.9 ± 12.6	31.2 (3.5)	30.5 ± 4.6	Obesity	400 mg	Capsules	HOMA-IR
Mielgo-Ayuso et al., 2014	Spain	Parallel	12 wk	43	40	19–49 years	33.7 ± 2.6	34.3 ± 3.0	Obesity	300 mg	Capsules	HOMA-IR, CRP
Brown et al., 2009	UK	Parallel	8 wk	46	42	52.15 ± 6.43/ 50.57 ± 6.48	31.21 ± 2.80	30.96 ± 2.45	Obesity	800 mg	Capsules	HOMA-IR
Cunningham et al., 2026	USA	Parallel	12 mo	50	47	60.4 ± 5.2 / 60.4 ± 5.5	29.1 ± 3.1	28.7 ± 2.7	Overweight	843 mg	Capsules	CRP
Dostal et al., 2016	USA	Parallel	12 mo	117	120	60.9 ± 0.45 / 60.6 ± 0.47	28.5 ±0.28	27.9 ± 0.25	Overweight and Obesity	1315 mg	Capsules	HOMA-IR
Bogdanski et al., 2012	Poland	Parallel	3 mo	28	28	49.2 ± 8.8 / 51.5 ± 7.4	32.5 ± 3.3	33.9 ± 2.3	Hypertension, Obesity	379 mg	Capsules	HOMA-IR, CRP
Nogueira et al., 2016	Brazil	Crossover	4 wk	20	20	41.1 ±8.4	33.56 ± 0.98	33.56 ± 0.98	Prehypertension, Obesity	1500 g	Capsules	CRP
Taghizadeh et al., 2017	Iran	Parallel	8 wk	25	25	32.2 ± 6.9/ 35.1 ± 7.2	32.3 ± 4.2	32.4 ± 5.9	Obesity	500 mg	Capsules	HOMA-IR
Mombaini et al., 2017	Iran	Parallel	45 d	22	23	23.22 ± 5.24 / 24.17 ± 6.83	28.96 ± 6.93	28.90 ± 4.30	PCOS, Overweight	500 mg	Tablets	CRP
Hussain et al., 2017	Pakistan	Parallel	12 wk	40	40	25 ± 18 / 28 ± 15	29.5 ± 2.2	28.6 ± 0.6	NAFLD, Overweight	1000 mg	Capsules	HOMA-IR, CRP

Study ID	Country	Design	Duration	N Int	N Ctrl	Age (years) (Int/Ctrl)	BMI Int (kg/m²)	BMI Ctrl (kg/m²)	Metabolic Status	Daily Dose	Formulation	Primary Outcome
Chen et al., 2016	Taiwan	Parallel	12 wk	39	38	44.1 ± 10.9 / 44.9 ± 11.9	31.0 (3.8)	30.0 ± 3.5	Obesity	856.8 mg	Capsules	HOMA-IR

Table S9. Summary of Included Studies: Omega 3. Int = Intervention; Ctrl = Control; NR = Not Reported; MetS = Metabolic Syndrome; IR = Insulin Resistance; NAFLD = Non-Alcoholic Fatty Liver Disease. All studies used placebo capsules or tablets as control. Data extracted directly from original publications, as reported, and units standardized.

Study ID	Country	Design	Duration	N Int	N Ctrl	Age (years) (Int/Ctrl)	BMI Int (kg/m²)	BMI Ctrl (kg/m²)	Metabolic Status	Daily Dose	Formulation	Control Type	Primary Outcome
H-Y Su et al., 2015	Taiwan	Parallel	12 wk	35	36	NR	29.3 ± 3.1	29.9 ± 4.6	MetS, Obesity	1280 mg EPA + 850 mg DHA	Capsules	Control diet	HOMA-IR
Krebs et al., 2006	UK	Parallel	24 wk	35	32	44.7 ± 13.2	35.0 (5.5)	35.0 ±5.5	IR, Obesity	1300 mg EPA + 2900 mg DHA	Capsule	Placebo	HOMA-IR, CRP
Nelson et al., 2007	USA	Parallel	8 wk	30	27	38.80 ± 11.97 / 38.15 ± 10.77	30.19 ± 5.50	30.46 ± 4.56	Obesity	11 g ALA	Capsule	Usual diet	HOMA-IR
Simão et al., 2012	Brazil	Parallel	90 d	19	15	47.9 ±9.98	32.93	35.36	MetS, Obesity	3000 mg n-3 (non specified)	Capsule	Usual diet	CRP, HOMA-IR
Schwab et al., 2018	Finland	Parallel	12 wk	18	20	58.9 ± 6.5	29.2 (2.4)	29.2 ±2.4	IR, Overweight/Obesity	10 g ALA	Oil	Control diet	HOMA-IR, CRP
Munro & Garg, 2012	Australia	Parallel	14 wk	17	12	40.5 ± 10.9 / 42.3 9.1	32.7 (3.28)	33.0 ± 3.17	Obesity	420 mg EPA + 1620 mg DHA	Capsule	Placebo	CRP
Chan et al., 2002	Australia	Parallel	6 wk	12	12	53.5 ± 9.0	33.6 ± 4.1	33.6 ± 4.1	Obesity	4 g n-3 (non specified)	Capsule	Placebo	HOMA-IR, CRP
Razny et al., 2015	Poland	Parallel	3 mo	24	24	47 ± 2	34.25 ± 0.7	35.24 ± 0.72	Obesity	1800 mg n-3 (DHA/EPA, 5:1)	Capsules	Placebo	HOMA-IR
de Luis et al., 2016	Spain	Parallel	6 mo	14	15	47.4 ± 9.1 / 44.3 ± 11.7	33.4 ± 1.4	32.95 ± 1.9	Obesity	500 mg n-3 (250 mg DHA)	Capsules	Placebo	HOMA-IR, CRP
Rajkumar et al., 2014	India	Parallel	6 wk	15	15	49 (Mean)	28.79	28.79	Overweight	180 mg EPA + 120 mg DHA	Capsules	Placebo	HOMA-IR, CRP
Simão et al., 2014	Brazil	Parallel	90 d	19	15	47.9 ± 9.98	34.3 ±7.2	36.3 ± 6.5	MetS, Obesity	1800 mg EPA + 1200 mg DHA	Capsules	Usual diet	HOMA-IR

Study ID	Country	Design	Duration	N Int	N Ctrl	Age (years) (Int/Ctrl)	BMI Int (kg/m²)	BMI Ctrl (kg/m²)	Metabolic Status	Daily Dose	Formulation	Control Type	Primary Outcome
Yang et al., 2019 (DHA+EPA)	China	Parallel	12 wk	35	34	55.09 ± 7.47 / 58.38 ± 6.87	26.92 ± 2.72	26.78 ± 2.98	Hypertension, Overweight	2000 g (DHA + EPA)	Capsules	Control oil	HOMA-IR
Yang et al., 2019 (ALA)	China	Parallel	12 wk	39	34	56.68 ± 7.83 / 58.38 ± 6.87	26.83 ± 3.06	26.78 ± 2.98	Hypertension, Overweight	2500 mg ALA	Capsules	Control oil	HOMA-IR
Derosa et al., 2012	Italy	Parallel	6 mo	78	79	≥ 18 and ≤ 75	26.0 ± 1.3	27.2 ± 1.9	Dyslipidemia, Overweight	3000 g n-3 (1200 mg EPA + 1350 mg DHA)	Capsules	Placebo	HOMA-IR, CRP
Tardivo et al., 2015	Brazil	Parallel	6 mo	44	43	55.1 ± 6.6 / 55.0 ± 7.3	32.8 ± 4.7	32.0 ± 4.6	MetS, Obesity	900 mg n-3 (non specified)	Capsules	Control diet	HOMA-IR, CRP
Qin et al., 2015	China	Parallel	3 mo	36	34	46.0 ± 10.68 / 44.3 ± 10.90	26.4 ± 3.9	26.0 ± 2.8	NAFLD, hyperlipidemia, Overweight	4 g "fish oil"	Capsules	Placebo	HOMA-IR
Shabrina et al., 2020	Taiwan	Parallel	12 wk	9	12	50.4 ± 11.2 / 43.4 ± 14.6	30.3 ± 3.74	31.8 ± 5.09	MetS, Obesity	1280 mg EPA + 850 mg DHA	Capsules	Control diet	HOMA-IR, CRP
Ebrahimi et al., 2009	Iran	Parallel	6 mo	47	42	53.5 ± 12.7 / 52.3 ± 11.1	30.3 ± 5.2	30.4 ± 6.1	MetS, Obesity	1000 mg fish oil (180 mg EPA + 120 mg DHA)	Capsules	Control diet	CRP
Mori et al., 2000 (EPA)	Australia	Parallel	6 wk	19	20	48.8 ± 1.1	29.0 ± 0.7	28.4 ± 0.5	Overweight	4000 mg EPA	Capsules	Placebo	HOMA-IR
Mori et al., 2000 (DHA)	Australia	Parallel	6 wk	17	20	48.8 ± 1.1	28.9 ± 0.7	28.4 ± 0.5	Overweight	4000 mg DHA	Capsules	Placebo	HOMA-IR
Albert et al., 2015	Australia	Crossover	8 wk	47	47	46.5 ± 5.1	27.4 ± 1.8	27.4 ± 1.8	Overweight	400 mg n-3 (230 mg EPA + 154 mg DHA)	Capsules	Placebo	HOMA-IR, CRP
Wong et al., 2013	Australia	Parallel	16 wk	13	12	60 ± 4	34 ± 2	33 ± 1	Obesity	4 g n-3 (non specified)	Capsules	Control diet	HOMA-IR

Study ID	Country	Design	Duration	N Int	N Ctrl	Age (years) (Int/Ctrl)	BMI Int (kg/m²)	BMI Ctrl (kg/m²)	Metabolic Status	Daily Dose	Formulation	Control Type	Primary Outcome
Huerta et al., 2016	Spain	Parallel	10 wk	16	21	20–50	27.5–40	27.5–40	Obesity	1300 mg EPA	Capsules	Placebo	CRP
Razny et al., 2021	Poland	Parallel	3 mo	13	14	41.0 ± 9.9	30.84 ± 3.48	31.56 ± 3.75	Obesity	1.8 g n-3 (non specified)	Capsules	Placebo	HOMA-IR
Jellema et al., 2004	Netherlands	Crossover	6 wk	11	11	59 ± 9	31.3 ± 1.82	31.3 ± 2.08	Obesity	1.35 g n-3 (600 mg EPA + 510 mg DHA)	Capsules	Placebo	HOMA-IR, CRP
