# Supplementary material for: Effects of Bioactive Compound Supplementation on Insulin Resistance and Systemic Inflammation in Overweight and Obese Adults: A Systematic Review and Meta-Analysis of Randomized Controlled Trials
Source: Nutrients. 2026 Oct 31;18(21):3638. doi: 10.3390/nu18213638 (PMC13765097; doi:10.3390/nu18213638)
Supplement: Supplementary file 1 [file nutrients-18-03638-s001.zip › Table S1. Detailed eligibility criteria of the systematic review.docx.pdf]

Table S1. Detailed eligibility criteria of the systematic review.

Domain	Inclusion criteria	Exclusion criteria
**Study design**	Randomized controlled trials (parallel or crossover), human studies, peer-reviewed full-text, Published in English-language peer-reviewed journals	Observational studies, non-randomized trials, animal/in vitro studies, reviews, conference abstracts, protocols
**Population**	Adults (≥18 years) with overweight or obesity (BMI ≥ 25 kg/m² or high waist circumference), without type 2 diabetes, With or without insulin resistance or metabolic syndrome	Diagnosed T2D, bariatric surgery patients, severe chronic disease affecting muscle metabolism, elite athletes
**Intervention**	Omega-3 PUFA, curcumin, resveratrol, or green tea extract supplementation; ≥4 weeks; no structured exercise intervention	Combined lifestyle interventions (exercise + supplement), protein supplementation trials, pharmacological agents, dietary pattern comparison, food matrices interventions
**Comparator**	Placebo, control supplement, control diet or usual diet; equivalent physical activity between groups	Studies where exercise differs between intervention and control arms.
**Outcomes**	Studies reporting at least one of the following:<br>Insulin sensitivity markers: HOMA-IR, Fasting insulin, Fasting glucose, OGTT, HbA1c.<br>Inflammatory biomarkers: CRP, IL-6, TNF-α, Adiponectin	Studies not reporting any predefined metabolic or body composition outcomes
**Language**	English	Non-English publications
**Publication type**	Full-text articles published in peer-reviewed journals.	Abstract-only publications

Abbreviations: BMI: body mass index; CRP: C-reactive protein; HbA1c: glycated hemoglobin; HOMA-IR: homeostatic model assessment of insulin resistance; IL-6: interleukin-6; OGTT: oral glucose tolerance test; PUFA: polyunsaturated fatty acids; T2D: type 2 diabetes; TNF-α: tumor necrosis factor alpha.

Nichele, Sarah; Prates, Lucas; Busseto, Luca. Effects of Bioactive Compound Supplementation on Insulin Resistance and Systemic Inflammation in Overweight and Obese Adults: A Systematic Review and Meta-Analysis of Randomized Controlled Trials.2026.
